# MAHRP2 is required for tether formation and cytoadherence in *Plasmodium falciparum* infected red blood cells

**DOI:** 10.3389/fcimb.2026.1675134

**Published:** 2026-05-29

**Authors:** Mohini A. Shibu, Gerald J. Shami, Eric Hanssen, Olivia M. S. Carmo, Vineet Vaibhav, Jumana Yousef, Laura F. Dagley, James McCarthy, Matthew W. A. Dixon

**Affiliations:** 1 Department of Infectious Diseases, The Peter Doherty Institute for Infection and Immunity, The University of Melbourne, Melbourne, VIC, Australia; 2 Infection and Global Health Division, Walter and Eliza Hall Institute, Melbourne, VIC, Australia; 3 Department of Biochemistry and Pharmacology, Bio21 Institute, The University of Melbourne, Melbourne, VIC, Australia; 4 Ian Holmes Imaging Centre, Bio21 Institute, The University of Melbourne, Melbourne, VIC, Australia; 5 Department of Medical Biology, The University of Melbourne, Melbourne, VIC, Australia; 6 Advanced Technology and Biology Division, Walter and Eliza Hall Institute, Melbourne, VIC, Australia

**Keywords:** exported proteins, MAHRP2, Maurer’s clefts, P*f*EMP1, tether

## Abstract

The ability of the human malaria parasite *Plasmodium falciparum* to remodel its host red blood cell (RBC) is central to the parasite’s ability to survive within the circulation of its host and cause disease. Following invasion, the parasite exports proteins into the RBC cytoplasm where they remodel the membrane skeleton and alter the cell’s biophysical properties. Many of the exported proteins are trafficked through parasite derived structures called Maurer’s clefts in the cytoplasm of the RBC. These clefts act as an intermediate compartment for proteins *en route* to the RBC membrane such as the major virulence protein, *Plasmodium falciparum* Erythrocyte Membrane Protein 1 (*Pf*EMP1). From the Maurer’s clefts, *Pf*EMP1 is delivered to the RBC membrane through a poorly understood process, which may involve trafficking via a physical connection and/or vesicle mediated trafficking. Electron-tomography of 3D7 infected RBCs reveals a tube-like structure, called the tether, that connects the Maurer’s clefts to the RBC membrane. To date, the formation and function of these tether structures is unclear, with only one protein, the Membrane Associated Histidine Rich Protein 2 (MAHRP2) known to locate at these structures. In this study, we show that deletion of MAHRP2 leads to a loss of tethers, reduced Maurer’s cleft immobilization at the RBC membrane and an increase in Maurer’s cleft size. Lastly, we show that deletion of MAHRP2 impacts PTP2 related vesicle formation, and a reduced ability to cytoadhere to the endothelial ligands CD36.

## 1 Introduction

Malaria is an infectious disease that causes more than 200 million infections and kills approximately 600,000 people every year (World Health Organisation, 2025). Accessed on 27/4/2026. *Plasmodium falciparum* malaria is the most virulent form and accounts for the majority of malaria related deaths. The virulence of *P. falciparum* malaria is associated with the adhesion of infected red blood cells (iRBCs) to the endothelial cells lining the capillaries in the body of the host. Adhesion is driven by the trafficking of the major virulence antigen *P. falciparum* Erythrocyte Membrane Protein 1 (*Pf*EMP1) to the RBC surface, which is facilitated by an orchestrated remodeling of the RBC by the parasite. This adherence of infected RBCs (iRBCs) in the microvasculature of the host allows the parasites to avoid immune surveillance mechanisms and splenic clearance (Barnwell, 1989).

Remodeling is facilitated by the export of at least 600 parasite proteins into the host cell cytoplasm which changes the biophysical properties of the RBC membrane and underpin the *de novo* assembly of parasite derived trafficking structures. This trafficking network consists of disc-shaped structures called Maurer’s clefts, electron dense vesicles (EDVs) (McMillan et al., 2013), chaperone complexes called J-dots (Külzer et al., 2010) and vesicle-like structures (VLS) (Tilley et al., 2008). All these structures have been shown to play roles in trafficking of *Pf*EMP1, with deletion of a number of different resident proteins resulting in a loss of parasite virulence (Maier et al., 2007; Maier et al., 2008; Spycher et al., 2008; Mundwiler-Pachlatko and Beck, 2013; Petersen et al., 2016; McHugh et al., 2020; Carmo et al., 2022). However, details of how the different components of the network work together to facilitate the final steps in *Pf*EMP1 trafficking from the Maurer’s clefts and its presentation in raised knob structures at the RBC membrane, remain unclear (Leech et al., 1984; Crabb et al., 1997).

In early-stage parasites, Maurer’s clefts are observed rapidly moving by Brownian motion within the host cytoplasm, becoming stationary around 22 hours post invasion (hpi) (Dixon et al., 2011). Maurer’s cleft immobilization coincides with the assembly of a network of host derived actin filaments (remodeled from the RBC) and the appearance of membrane structures called tethers which together appear to connect the Maurer’s clefts to the membrane skeleton (Cyrklaff et al., 2011; Cyrklaff et al., 2012). Studies investigating the role of actin in this process has shown that disassembly of the filaments leads to only a small change in Maurer’s cleft immobilization (Kilian et al., 2013), suggesting that another tethering mechanism is at play.

Electron tomography studies of 3D7 trophozoite iRBCs revealed these tether structures as rod-like extensions that are ~30 nm in diameter and ~100 nm long that appear to be connected to the periphery of the Maurer’s clefts as well as the cytoplasmic side of the RBC membrane (Hanssen et al., 2008). The only known protein that localizes to these tethers is the Membrane Associated Histidine Rich Protein 2 (MAHRP2, PF3D7_1353200). Previous, fluorescence microscopy studies have shown that MAHRP2 is expressed early in development (from 2 hpi) and appears to locate adjacent to the Maurer’s clefts throughout development (McMillan et al., 2013). Previous attempts to delete *mahrp2* using conventional knockout approaches have proven unsuccessful, suggesting that the protein may play an essential role in parasite development (Pachlatko et al., 2010). In this study we use a combination of super resolution microscopy and immuno electron microscopy (IEM) to observe and describe the different architectures of the Maurer’s clefts and tether structures in 3D7 and NF54 iRBCs, showing that 3D7 clefts appear smaller and more condensed throughout development while NF54 clefts are larger and have a more prominent disk-like structure later in parasite development. We also show that NF54 parasites have a more spherical tether structure than the classical tethers described in 3D7 parasites previously (Pachlatko et al., 2010). We show that cKO of MAHRP2 leads to a loss of tether structures, a reduction in cleft immobilization at the RBC membrane and an increase in Maurer’s cleft size. Lastly, we show that the deletion of MAHRP2 has a global effect on exported proteins that locate at the Maurer’s cleft, J-dots and vesicles, which results in a significant reduction in iRBC cytoadhesion to the endothelial ligand CD36.

## 2 Materials and methods

### 2.1 Parasite culture

*Plasmodium falciparum* parasites were cultured in O+ human erythrocytes (Lifeblood, Australia) at 5% haematocrit in RPMI-1640 media with GlutaMAX and HEPES (Gibco, ThermoFisher) (Trager and Jensen, 1976). The RPMI was supplemented with 0.25% (w/v) AlbuMAX II (Gibco, ThermoFisher), 5% (v/v) pooled human serum (Lifeblood, Australia), 10 mM glucose, 0.5 mM hypoxanthine and 42 μM gentamicin (Sigma-Aldrich, Merck) to support growth. Cultures were maintained at 37 °C in a gas-tight microisolator box gassed with malaria mix (1% O_2_, 5% CO_2_, 94% N_2_). Parasitaemia was calculated from Giemsa (10% v/v) stained thin smears of parasite infected RBCs (iRBCs) visualized under a 100x objective with immersion oil. The 3D7-MAHRP2-GFP cell line previously described in McMillan et al, 2013 was used in this study and was maintained on 2.5 nM WR99210 (McMillan et al., 2013).

Synchronized ring stage iRBCs were prepared as required by selectively lysing mature-stage iRBCs with 5% (w/v) D-sorbitol (Sigma-Aldrich, Merck) (Lambros and Vanderberg, 1979). Mature stage iRBCs were purified from mixed stage cultures by either magnetic separation using CS MACS columns or by density separation on a 65% Percoll gradient (Kramer et al., 1982). Trophozoite parasites were subjected to Gelatine flotation using 70% (v/v) Gelofusine^®^ (Braun) as previously described to maintained knob-positive parasites (Goodyer et al., 1994).

### 2.2 Plasmid constructs and transfections

To generate the MAHRP2-HA cKO parasite line, a DNA fragment was synthesized by Genewiz consisting of a 5’ homology region (-444 – 6bp), an artificial intron containing a *loxP* site and a recodonised version of the 3’end of the *mahrp2* locus (32 – 534bp). The guide and PAM sequence was also recodonised. This sequence was directionally cloned into the NotI and PstI sites in the pGLMS-loxP-HA plasmid (Genewiz). A 3’ homology region was PCR amplified from gDNA using the MAHRP2 HR2 F and MAHRP2 HR2 R primers (**Supplementary Figure 1C**) and directionally cloned into the EcoRI and KasI sites of the plasmid. The resulting pGLMS-loxP-MAHRP2-HA repair plasmid was then prepared for transfection by linearizing the plasmid. The CRISPR guide sequence (TTGTATACATCATATGGACA) was selected using CHOP-CHOP software and ordered as an Alt-R CRISPR-Cas9 crRNA (IDT™). The guide was complexed with the Cas9 recombinant protein following the manufactures recommendations immediately before transfection. Ring-stage parasites were transfected with 100 μg of linearized repair plasmid mixed with the complexed guide by electroporation as previously described (Rug and Maier, 2013). A NF54 parasite line containing the rapamycin (Rapa) inducible split diCRE cassette was used as the parental line (Wilde et al., 2019). Parasites in which the plasmid had integrated were selected by the addition of WR99210 treatment. Correct integration was verified by PCR using the MAHRP2 intF and HA R or MAHRP2 intF and glmS R primer pairs (**Supplementary Figures 1A, B**).

### 2.3 Conditional knockout of MAHRP2

The MAHRP2-HA parasite line contains *loxP* sites which can facilitate diCRE mediated conditional KO upon the addition of Rapa. Following the addition of Rapa, the FKBP12 and FRB proteins fused to the two enzyme halves of the split CRE dimerize creating a functional CRE protein which recognized the *loxP* sites and deleted the sequences between them. To induce cKO in the MAHRP2-HA parasites, synchronized schizont stage iRBCs were incubated with 100 nM Rapa or the equivalent volume of DMSO as a control and harvested ~30h later in the next asexual cycle when they reached trophozoite stage of development.

### 2.4 Parasite growth assay and flow cytometry

Parasite cultures were tightly synchronized to a 2-hour window and returned to culture. The ring stage iRBC culture was diluted to 1% haematocrit, 0.25% parasitaemia and used to seed a 24-well plate. This denotes the start of cycle 1 of the assay. DMSO and Rapa treatment condition were investigated from triplicate wells for each experiment. Three experiments were performed. The parasitaemia was determined by flow cytometry of trophozoite samples collected at cycles 1, 2 and 3. For flow cytometry analysis 150 μL of culture from each well of the 24 well plate was placed in a 1.5 mL tube containing 1ml of PBS. The sample was centrifuged (500 x g for 5 min) and the pellet resuspended in 0.125% glutaraldehyde/PBS and incubated for 20 min at room temperature. The fixed iRBCs were washed thrice in PBS (with centrifuging between washes). The iRBC pellet was resuspended in a solution of SYBR Green in PBS (1:4000 dilution) and incubated at room temperature for 20 mins. Following staining the iRBCs were again centrifuged and washed in PBS and diluted to 0.1% haematocrit before analyzing. Flow cytometry was performed on a Attune NxT Flow Cytometer (Thermo Fisher), 100,000 events were recorded per well. Data analysis was performed using Flow Jo software.

### 2.5 Protein sample preparation and immunoblotting

Infected RBCs were harvested from culture by centrifugation at 500 x g for 5 min. The iRBCs were lysed by resuspending in 10 volumes of ice cold 0.03% saponin (Sigma-Aldrich, Merck) in PBS and incubated on ice for 10 min. Lysates were centrifuged at 2,000 × g for 5 min at 4˚C. The supernatant was discarded and the pellet washed thrice in PBS containing cOmplete EDTA-free Protease Inhibitor Cocktail (Roche). The washed pellet was resuspended in PBS and mixed with 4X LDS buffer, 10X DTT (NuPAGE, ThermoFisher) and heated at 70˚C for 10 min. Samples were separated by SDS-PAGE on either 4-12% Bis-Tris or 3-8% Tris-Acetate gradient gels (NuPAGE, ThermoFisher) run in MES, MOPS, or Tris-Acetate SDS running buffers (NuPAGE) as per the manufacturers recommended conditions. Protein gels were either dry transferred onto nitrocellulose membranes using the iBlot 2 device (Invitrogen) or wet transferred onto PVDF membrane using the Mini Trans-Blot Cell (Bio-Rad). Wet transfers were conducted overnight at 4˚C with ice cold transfer buffer (44 mM Tris, 30 mM glycine, 0.03% SDS, 20% v/v methanol) at 20–30 V. Membranes were blocked in 5% w/v skim milk in PBS with 0.5% Tween20 (PBS-T) for at least 1h at room temperature.

The primary and secondary antibodies were diluted in 5% skim milk- PBS-T. Primary antibodies used include mouse αHA (1:2000, Sigma), rabbit αERC [1:2000 (Parkyn Schneider et al., 2017)], rabbit αREX1-rpt [1:2500 (Hawthorne et al., 2004)], rabbit αSBP1 [1:2500 (Cooke et al., 2006)], mouse αMAHRP1c [1:2500 (Spycher et al., 2008)], and anti-ATS [1:250 (Cooke et al., 2006)]. Membranes were probed with the relevant primary antibody for either 1h at room temperature or overnight at 4˚C. Membranes are washed 3 times in PBS-T and probed with either anti-mouse or rabbit HRP conjugated secondary antibodies (1:5000, Merck AP181P, AP132P). After primary and secondary antibody incubation membranes were again washed 3 times in PBS-T before being incubated with Clarity ECL reagents (Bio-Rad) or SuperSignal West Femto Maximum Sensitivity Substrate and visualized on a ChemiDoc Imaging system (BioRad). Protein bands were quantified using FIJI ImageJ software (Schindelin et al., 2012) by defining a region of interest and measuring mean grey value (Davarinejad).

### 2.6 *Pf*EMP1 trypsin cleavage assay

Cultures containing at least 6% mid-trophozoite stage-iRBCs were enriched using Percoll or magnetic separation. iRBCs were harvested at 300 × g for 5 min and split into 3 separate tubes containing either 10 volumes of warm 1xPBS (P) or 1xPBS containing 1 mg/ml TPCK-Treated Trypsin (Sigma-Aldrich, Merck) (T) or 1xPBS containing 1 mg/ml TPCK-Treated Trypsin (Sigma-Aldrich, Merck) pre-incubated with trypsin inhibitor from Glycine max (5 mg/ml) (Sigma) (i). Samples were incubated at 37˚C for 1 h, prior to the addition of trypsin inhibitor (5 mg/ml) to the P and T samples and incubated for 15 min at room temperature. The samples were then centrifuged at 300 x g for 5 min. The pellet fraction was solubilized in 10 volumes of ice cold 1% TritonX-100 (Sigma-Aldrich) in PBS containing cOmplete EDTA-free Protease Inhibitor Cocktail (Roche) and incubated on ice for 30 min. All subsequent steps contained 1x cOmplete EDTA-free Protease Inhibitor Cocktail (Roche). Samples were centrifuged at 16,000 × g for 10 min at 4˚C and then the pellets were washed three times in the ice cold TritonX-100 solution. The pellet was solubilized in 20 volumes of 2% SDS/PBS and mixed at room temperature for 30 min. The samples were centrifuged at 16,000 × g for 10 min and the supernatant transferred to a new tube and prepared for SDS-PAGE and Western blotting (section 3.5). Mouse anti-ATS antibodies are used to detect *Pf*EMP1 and rabbit αSBP1 is used as a loading control (Maier et al., 2007; Maier et al., 2008).

### 2.7 Live-cell fluorescence microscopy

Two microliters of iRBCs were applied on a slide and flattened with a cleaned coverslip and the coverslip seated to the slide using nail polish prior to imaging. The slide was imaged immediately. For membrane staining, iRBCs were washed and resuspended in 1xPBS and stained with 1 μM BODIPY-TR-ceramide in PBS (Thermo Fisher) for 2hrs. The stained iRBCs were washed with 1xPBS prior to imaging on a DeltaVision Elite restorative widefield deconvolution imaging system (GE Healthcare). The filter set used was tetramethyl rhodamine isocyanate (TRITC) (excitation [Ex] 542/27, emission [Em] 594/45 nm wavelengths). A 100X UPLS Apo 1.4 NA oil immersion objective (Olympus) was used for imaging.

### 2.8 Immunofluorescence assays

Infected RBCs were washed in 1xPBS and resuspended at 50% haematocrit in PBS. Coverslips were coated with 0.01 mg/ml *Phaseolus vulgaris* erythroagglutinin (PHA-E) lectin (Sigma-Aldrich, Merck) and incubated at 37˚C for 15–30 min. The washed iRBCs were overlaid on the lectin coated coverslips to allow adhering for 15 min at room temperature. The unbound cells are washed off with 1 x PBS and the sample fixed with 4% formaldehyde (Thermo Scientific), 0.0065% glutaraldehyde (Thermo Scientific) in PBS for 20 min. The samples were then washed 3 times in PBS and permeabilized with 0.1% (v/v) TritonX-100 (ChemSupply) for 10 min. The samples were washed three times in PBS. Primary antibodies diluted in 3% BSA were added to the wells for 1h at room temperature. Primary antibodies used include rabbit αREX1-rpt [1:1000 (Hawthorne et al., 2004)], mouse αKAHRP 89 (1:1000), mouse αHA (1:500, Sigma), rabbit αHA (1:500), rabbit αSBP1 [1:500 (Cooke et al., 2006)], mouse αMAHRP1c [1:500 (Spycher et al., 2008)], rabbit αPTP2 [1:200 (Maier et al., 2008)], rabbit αGFP [1:500 (Humphries et al., 2005)], mouse αGFP (1:500, Roche), mouse αHSP70x (1:500) and chicken αGFP (1:500, Sigma). The wells were washed with PBS and the secondary antibodies diluted with 3% BSA was added for 1h at room temperature. Secondary antibodies used include rabbit Alexa Fluor 568 (A11036, Invitrogen), rabbit Alexa Fluor 647 (A21245, Invitrogen), mouse Alexa Fluor 488 (A11029, Invitrogen), mouse Alexa Fluor 594 (A11005, Invitrogen) and mouse Alexa Fluor 647 (A21236, Invitrogen). The wells were again washed in PBS, stained with 4’,6-diamidino-2-phenylindole (DAPI) (2 μg/mL), then mounted with anti-fade made of 5 mM p-phenylenediamine (PPD) (Sigma-Aldrich, Merck) in 90% glycerol. Coverslips were sealed onto the glass slides using nail polish and were stored at 4 °C prior to imaging.

Super-resolution confocal microscopy was performed on the LSM880 with Airyscan (Zeiss). Samples were imaged using a 63x Plan-Apochromat (Zeiss, 1.4 NA) oil immersion lens. The following Elyra laser emission and band pass filter sets were used: GFP (Ex488, Em bandpass 495-550 + lowpass 570 nm), RFP (Ex561, Em bandpass 420+ lowpass 495–620 nm), Cy5 (Ex642, Em bandpass 570-620 + lowpass 645 nm).

Images were processed using FIJI ImageJ software and deconvolved with Huygens image analysis software (Professional version 19.04, Scientific Volume Imaging). Colocalization analysis was performed using JACoP, a Pearson’s coefficient plug-in on FIJI (Bolte and Cordelières, 2006).

### 2.9 Expansion microscopy

Expansion microscopy was performed on trophozoite stage infected RBCs by following published protocols (Bertiaux et al., 2021; Liffner and Absalon, 2021). 12mm diameter coverslips (#1.5, Hurst Scientific) were placed in 24-well plates; poly-D-Lysine (Gibco) was added to these coverslips and incubating for 1hr at 37 °C. Synchronous parasite culture was added to the poly-D-Lysine coated coverslip and incubate for 30 min at 37 °C. The unbound parasites were gently washed from the coverslips and the slide fixed with 4% paraformaldehyde in PBS for 20 mins. Next protein crosslinking was performed by adding 1.4% paraformaldehyde/2% acrylamide solution in PBS onto the fixed sample and incubating overnight at 37 °C. Gelation of the sample was performed by adding tetraethylenediamine (final 0.5%) and ammonium persulfate (final 0.5%) to a monomer solution (19% sodium acrylate (Sigma-Aldrich, Merck), 10% acrylamide and 0.1% of N,N’-methylenebisacrylamide (Sigma-Aldrich, Merck) in PBS) prepared at least a day prior. This mix was placed on squares of parafilm on ice and the washed coverslip quickly flipped cell-side down onto the monomer solution and incubated for 5 mins on ice, followed by 30 mins at 37 °C. The coverslips with gel was then placed into denaturation buffer (200mM SDS, 200mM NaCl, 50mM Tris in water, pH 9) for 15 mins at room temperature while shaking. The gel was then separated from the coverslip placed in denaturation buffer and incubated at 95 °C for 90 mins. Once cooled the gel was placed in water for the first round of expansion. On expansion the diameter of the gels were measured to calculate the expansion factor. Next the gels were shrunk by washing in PBS 3 times with 15 min incubations before the addition of antibodies. Primary antibodies were diluted in 3% BSA in PBS and incubated overnight. The gels were washed three times with PBS-T. Secondary antibodies and N-Hydroxysuccimide-Ester (AF 594 NHS-ester (Lumiprobe) were diluted in PBS and added to the sample and incubated for 2.5 hr at room temperature. The gels were washed again with PBS-T. The gels were then expanded a final time with three washes with water on a shaker with 15 min incubations. Sections of the expanded gel were cut and mounted on poly-D-Lysine coated coverslips (25mm diameter) to image. Primary and secondary antibodies used are detailed above.

Imaging was performed on the LSM880 with Airyscan (Zeiss) using the filter settings described above. Samples were imaged using a 63x Plan-Apochromat (Zeiss, 1.4 NA) water immersion lens.

### 2.10 *Pf*EMP1 binding assay under flow conditions

Cytoadhesion binding assays under physiological flow conditions were performed in ibidi μ-Slide 0.2 channel slides. The channels were coated with 125 μg/mL recombinant human CD36 (R&D Systems) and incubated overnight at 4 °C. The CD36 solution was then replaced with 1% BSA/1xPBS and incubated for 1 h at room temperature. Validation of the specificity of this assay has shown that iRBC do not non-specifically bind to BSA coated slides or other non-relevant proteins.

When ready to perform an experiment the BSA solution was gently flushed from the channel with warm bicarbonate-free RPMI 1640 (Invitrogen) and placed in the DeltaVision Elite widefield imaging system (GE Healthcare) with a 37 °C environment chamber. Synchronized trophozoite stage parasites (2–4 hpi window, 3% parasitemia and 1% haematocrit) were resuspended in warm bicarbonate-free RPMI 1640 and were pulled through the channel at 100 μl/min for 10 min at 37 °C using a programmable syringe pump (Harvard Apparatus) to allow cytoadherence. The channel was then washed for 10 min with bicarbonate free RPMI at the same flow rate to remove unbound cells. After 10 mins, still under flow, the adhered cells were counted at 10 pre-programmed points across the channel. The cells were observed using the 63X objective 1.4 NA (Olympus).

### 2.11 Scanning electron microscopy

Trophozoites were magnet purified and fixed in 0.05% glutaraldehyde in 0.1M Sorensens Phosphate Buffer (SPB) pH 7.4 for 20 min prior to fixation in 2.5% glutaraldehyde in 0.1 M SPB for 30 min at room temperature. Cells were washed in 0.1 M SPB and incubated in 1% osmium tetroxide (w/v) for 30 min. Cells were washed in ultrapure water and dehydrated via sequential 5 min incubations in 30%, 50%, 70%, 80%, 90%, 95%, and (3x) 100% ethanol followed by critical point drying in a Leica CPD300 critical point dryer. Immediately prior to imaging, samples were coated with 5 nm gold with a Safematic CCU-010 sputter coater. Images were acquired with the Everhart-Thornley detector in ‘Optiplan’ mode of an FEI Teneo SEM in using at a working distance of 5 mm, a beam current level of 50 pA, and 2 kV accelerating voltage.

### 2.12 Immuno electron microscopy

iRBCs (30–60 mL of ~5% parasitemia at 24–32 hpi) were harvested by magnet purification and fixed in 10x pellet volumes of 2% (v/v) paraformaldehyde (PFA)/PBS for 20 min at room temperature. Cells were washed in PBS and then permeabilized using 10x pellet volume of 1 haemolytic unit (HU) of Equinatoxin II for 6 min at room temperature. Cells were washed and briefly fixed again in 2% PFA/PBS for 5 min and washed again. For immunogold labelling, cells were blocked using 3% BSA/PBS for 1h and then incubated in two pellet volumes of primary antibody (1:10 diluted in 3% BSA/PBS) for 2 h. Primary antibodies used for electron microscopy include mouse αHA (Sigma), rabbit αGFP (Humphries et al., 2005), mouse αbeta-tubulin (Sigma) and rabbit αPTP2 (Maier et al., 2008). Cells were washed thrice with PBS and incubated in one pellet volume of the gold secondary antibody for 1 h (1:15; Aurion protein-A EM-grade 6-nm diameter gold; catalogue no. JA806-111). Cells were washed in 3% BSA/PBS and then in PBS. Cell pellets were fixed in 2.5% glutaraldehyde at 4 °C for storage prior to embedding.

Cells were then fixed in 1% osmium tetroxide (w/v) in 0.1 M cacodylate buffer or SPB (Sorensen’s Phosphate Buffer, Na_2_HPO_4_/NaH_2_PO_4_pH 7.4) for 30 min at room temperature in darkness. Cells were washed with 0.1 M cacodyalate or SPB and incubated in 1% tannic acid (w/v) in 0.1 M SPB for 20 min. Cells were washed in ultrapure water and dehydrated in an ascending concentration series of ethanol and acetone. Samples were progressively infiltrated with Procure 812 Epon-substitute resin and polymerized at 60 °C for 48 hours. Ultrathin (70 nm) and semithin (250 nm) sections were generated using a Leica EM Ultracut 7 ultramicrotome (Leica, Heerbrugg, Switzerland) and post-stained using 2% uranyl acetate and Reynold’s Lead Citrate (8 µM lead nitrate, 136.4 µM sodium citrate, 160 µM sodium hydroxide) for 10 min each, respectively. Transmission electron microscopy was performed on an FEI Tecnai F30 electron microscope (FEI Company, Hillsboro, OR) at an accelerating voltage of 200 kV and Talos L120C at an accelerating voltage of 120kV.

### 2.13 Whole cell proteomics

Four biological replicates of control and MAHRP2 cKO iRBCs were analyzed. 30–60 mL of iRBCs (20–32 hpi, ~5% parasitemia) were harvested by magnet purification and lysed using 10x pellet volumes of 0.03% saponin/PBS for 10 min on ice. The solution was centrifuged at 16, 000 x g, the supernatant discarded, and the pellet washed at least three times in 1x PBS. The pellets were lysed in 100 µL of preheated (95°C) buffer (2.5% SDS in 100 mM Tris-HCl, pH 8.5). Next DNA was hydrolyzed with the addition of 2 µL neat TFA (Sigma) and lysates were neutralized to pH 8.5 by the addition of 40 µL 1M Tris-HCl as previously described (Dagley et al., 2019). Protein concentrations were determined using Pierce™ BCA Protein Assay Kit following manufacturers’ instructions. Cell lysates (20 µg protein per replicate) were transferred to 0.5 mL LoBind Deep Well plate (Eppendorf) prepared for mass spectrometry analysis using the modified SP3 protocol (Hughes et al., 2019), with some modifications. Briefly, samples were subjected to simultaneous reduction and alkylation with a final concentration of 10 mM Tris (2-carboxyethyl) phosphine (TCEP) and 40 mM 2-chloracetamide followed by heating at 95 °C for 10 mins. Prewashed magnetic PureCube Carboxy agarose beads (20 µL, Cube Biotech) were added to all the samples along with acetonitrile (ACN,70% v/v final concentration) and incubated at room temperature for 20 min. Samples were placed on a magnetic rack and supernatants were discarded, and beads were washed twice with 70% ethanol and once with neat ACN. ACN was completely evaporated from the tubes using a CentriVap (Labconco) before the addition of digestion buffer (50 mM Tris-HCl, pH 8) containing 1 µg each of enzymes Lys-C (Wako, 129–02541) and SOLu-Trypsin (Sigma-Aldrich, EMS0004). Trypsin-LysC on-bead digestion was performed with agitation (400 rpm) for 1 h at 37 °C on a ThermoMixer C (Eppendorf). Following digestion, the samples were transferred to pre-equilibrated C18 StageTips (2× plugs of 3M Empore resin, no. 2215) for sample clean-up. The eluates were lyophilized to dryness before being reconstituted in 0.1% FA/2% ACN ready for mass spectrometry analysis.

Reconstituted peptides were separated using reverse-phase liquid chromatography on a 15 cm C18 fused silica column with an integrated emitter tip (IonOpticks, ID 75 µm, OD 360 µm, 1.6 µm C18 beads) on a NeoVanquish LC coupled to Thermo Orbitrap Astral. Peptides were analyzed on a 25 min linear analytical gradient of increasing buffer B (80% ACN, 0.1% FA) from 2 to 34%. Data was acquired in a data independent (DIA) mode. The MS1 settings were as follows: Orbitrap resolution 240,000; scan range m/z 380-980; AGC target 500%. The DIA parameters were as follows: isolation window: custom; HCD collision energy: 25%; scan range: m/z 145-1450; maximum injection time: 3 ms; AGC target: 800%.

Raw DIA data were analyzed on Spectronaut (Bruderer et al., 2015) version 19.2 using BGS factory settings of directDIA analysis. *Plasmodium falciparum* Uniprot Reference Proteome (2021) was used for database searching.

Data processing and analysis were performed using R (version 4.4.1). Proteins without any proteotypic precursors, with q-value greater than 0.01 and/or were identified by one peptide were removed. In addition, only proteins that were quantified in at least 50% of replicates in at least one condition were kept. Total of 4,219 proteins were included in the analysis. Protein intensities were log_2_-transformed (Hediyeh-Zadeh et al., 2023). No imputation was applied, except for proteins that were completely missing in one group but detected in the other group, to enable statistical comparison. For these proteins, missing values were imputed using random numbers drawn from a normal distribution shifted 1.8 standard deviations down and with a width (standard deviation) of 0.3. The data was normalized using RUVIIIC (v.1.0.19). Differential analysis was performed using limma (v.3.52.4). A protein was determined to be significantly differentially expressed if the absolute value of log fold change was ≥ 1 and the false discovery rate (FDR) was ≤ 5% after correcting for multiple testing using Benjamini–Hochberg (H-B) approach. The mass spectrometry proteomics data have been deposited to the ProteomeXchange Consortium via the PRIDE (Perez-Riverol et al., 2025) partner repository with the dataset identifier PXD065129.

## 3 Results

### 3.1 MAHRP2 labelled tethers have a different morphology in NF54 parasites

To characterize MAHRP2, we tagged the endogenous locus with a 3xHA tag at its 3’ end and incorporated flanking *loxP* sites into the sequence (**Supplementary Figure 1A**). A NF54 parental line that stably expresses a rapamycin (Rapa) inducible split CRE was used allowing for conditional knockout (cKO) of MAHRP2. Following transfection and the recovery of parasites, the correct integration of the plasmid into the *mahrp2* locus was verified by PCR (**Supplementary Figures 1B–D**).

**Figure 1:**
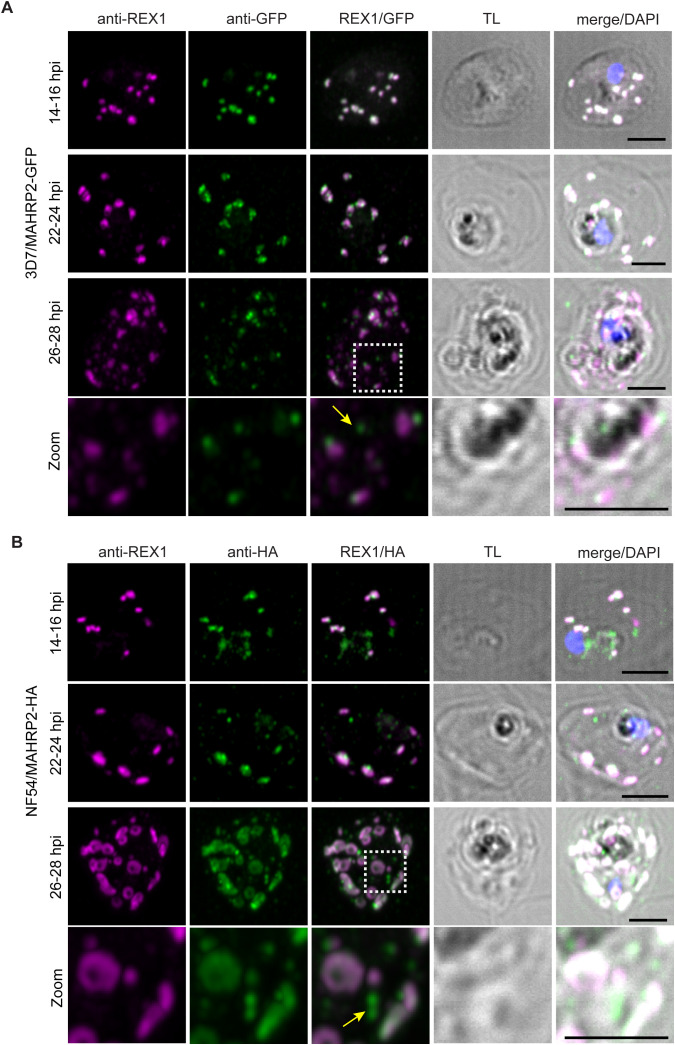
Localisation of MAHRP2-GFP and MAHRP2-HA through development. **(A)** Super-resolution imaging of 3D7-MAHRP2-GFP parasite (green) co-stained with antibodies to the Maurer’s clefts marker REX1 (magenta) at the timepoints 14-16 hpi (rings), 22–24 hpi and 26–28 hpi. Zoomed images show distinct MAHRP2 puncta in the cytoplasm independent of the Maurer’s clefts in 26–28 hpi iRBC. **(B)** Super-resolution imaging of NF54-MAHRP2-HA parasite (green) co-stained with antibodies to REX1 (magenta) at the timepoints 14-16 hpi (rings), 22–24 hpi and 26–28 hpi. Zoomed images of 26-28 hpi iRBC show distinct MAHRP2 puncta in the cytoplasm independent of the Maurer’s clefts. (yellow arrows) TL – transmitted light. DAPI stains the parasite nucleus. Merged images of all channels are shown. Scale: 2 µm.

To gain an understanding of the pattern of localization of MAHRP2 in NF54 across parasite development we performed Airyscan super resolution microscopy of NF54-MAHRP2-HA iRBCs and compared them to a previously generated 3D7-MAHRP2-GFP parasite line (McMillan et al., 2013). Infected RBCs were tightly synchronized and IFAs performed at 14-16, 22–24 and 26-28 hpi (**Figures 1A, B**). MAHRP2 was localized using anti-GFP or anti-HA antibodies and the clefts were labelled with anti-REX1 antibody (Hawthorne et al., 2004). MAHRP2-HA staining is observed at the REX1 labelled Maurer’s clefts in NF54 iRBC at each timepoint examined (**Figures 1A, B**). However, in contrast to 3D7 we see a more pronounced and larger disk-like Maurer’s cleft at the 26-28 hpi timepoint in NF54 parasites (**Figures 1A, B**). While the location of MAHRP2 is similar in 3D7 and NF54 early in development, MAHRP2 labelling of the cleft is more pronounced and more MAHRP2 labelled puncta are present in the NF54 iRBCs compared to 3D7 iRBCs later in development (26-28 hpi) (**Figures 1A, B**; yellow arrows). These differences warranted further investigation.

**Figure 2:**
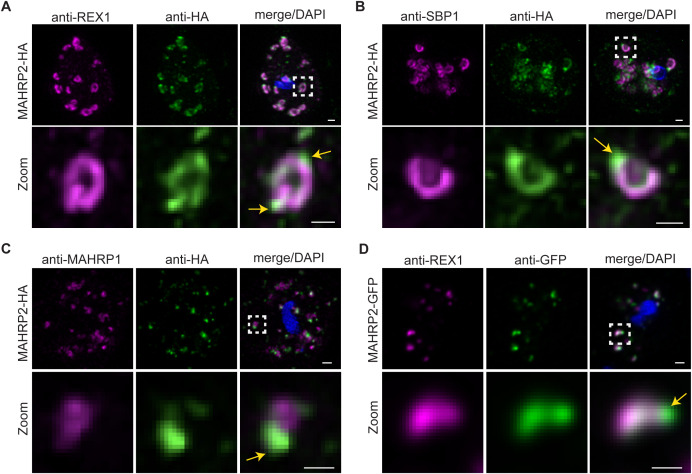
MAHRP2 locates to the outer compartment of Maurer’ clefts. **(A–C)** Super-resolution imaging of a MAHRP2-HA parasite (green) co-stained with antibodies to the outer Maurer’s clefts markers REX1 (**A,** magenta) and SBP1 (**B**, magenta), and inner compartment marker MAHRP1 (**C**, magenta). **(D)** Super-resolution imaging of a MAHRP2-GFP parasite (green) co-stained with antibodies to the outer Maurer’s clefts marker REX1 (magenta). Zoomed images of single Maurer’s clefts showing accumulation of MAHRP2 at distinct points (yellow arrows) on the outer compartment. DAPI stains the parasite nucleus. Merged images of all channels are shown. Scale: 500 nm.

Prior studies using super resolution microscopy have shown that the Maurer’s clefts of mature trophozoites (>26-28 hpi) exhibit distinct sub-compartments, with different resident proteins present in different sub-compartments (McMillan et al., 2013; McHugh et al., 2020). It was shown that both REX1 and SBP1 are concentrated at the periphery of the Maurer’s cleft, while MAHRP1 is concentrated in the central part of the cleft (McMillan et al., 2013; McHugh et al., 2020). In co-location experiments NF54-MAHRP2-HA signal is associated with one or two distinct puncta at the periphery of the Maurer’s clefts as previously described, with additional signal overlapping with REX1 and SBP1 at the cleft periphery and minimal overlap with MAHRP1 (**Figures 2A–C**; **Supplementary Figures 2A–C**). Image analysis reveals a high level of colocation of MAHRP2 with REX1 (Pearson’s coefficient (r), 0.83) and SBP1 (0.69) at the periphery of the Maurer’s cleft disc. In contrast, MAHRP2 shows very little overlap with MAHRP1 (r = 0.25) (**Supplementary Figures 2E, F**).

**Figure 3:**
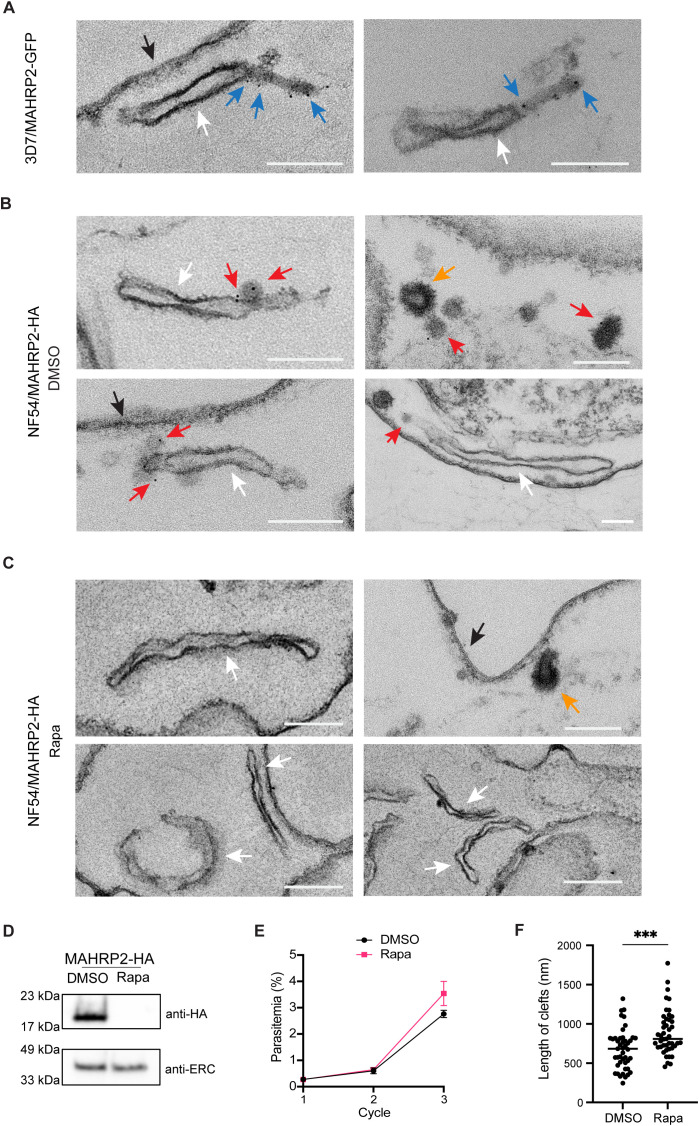
Deletion of MAHRP2 leads to a loss of tethers and alters Maurer’s cleft architecture. **(A)** Immuno-EM of 3D7-MAHRP2-GFP with anti-GFP/gold bead labelling. Blue arrows indicate the anti-gold bead labelled rod-like electron dense structures associated with the Maurer’s clefts. Scale bar: 200nm. **(B)** Immuno-EM of NF54-MAHRP2-HA DMSO treated iRBC with anti-HA/gold labelling. Red arrows indicate the anti-gold bead labelled vesicle-like structures found associated with the Maurer’s cleft and in the iRBC cytoplasm. Scale bar: 200 nm. **(C)** Immuno-EM of NF54-MAHRP2-HA Rapa treated iRBC with anti-HA/gold bead labelling. No gold labels or tethers are observed. Maurer’s clefts – white arrows, RBC membrane – black arrows and EDVs – orange arrows. Scale: 200 nm. **(D)** Western blot of protein extracts from DMSO and Rapa treated parasites. Blots were probed with anti-HA showing a loss of protein following deletion. Anti-ERC antibodies were used as a control. **(E)** Parasite growth assay measuring parasite replication over 3 asexual cycles. Rapa was added at the start of cycle 1 during ring stage development and growth was assessed by sampling parasites at trophozoite stages in cycles 1, 2 and 3, staining with SYBR-Green and measuring parasitemia by flow cytometry (n = 3 biological replicates). Graph shows mean parasitaemia and standard deviation. MAHRP2 cKO cells show a small but non-significant (p = 0.18) growth advantage after 3 cycles. **(F)** Length of Maurer’s clefts in DMSO (678 ± 285 nm) and Rapa (891 ± 234 nm) treated iRBCs were measured and plotted from thin-section electron micrographs. n = 45 clefts, 3 biological repeats, unpaired t-test p = 0.0003***. The mean values ± SEM are plotted.

Imaging of the 3D7-MAHRP2-GFP parasite line confirmed that MAHRP2 co-locates with REX1 at the Maurer’s cleft periphery (r = 0.87) and is concentrated at certain points of the cleft (**Figure 2D**, **Supplementary Figure 2D**). This data demonstrates that MAHRP2 locates to the cleft periphery in addition to exclusive staining of the certain regions of the clefts. To confirm this localization pattern and to visualize the ultra-structure of the tethers in NF54 we employed immuno-electron microscopy (immuno-EM). Thin section transmission electron microscopy (EM) imaging of the 3D7-MAHRP2-GFP reveals electron dense rod-like structures associated with the Maurer’s clefts that label with anti-GFP/gold with the density and positioning of the labelling equivalent to previously published data (Pachlatko et al., 2010) (**Figure 3A**, blue arrows). In contrast to 3D7, no classical tether-like structures were observed in the NF54 line, instead we see rounder fuzzier structures attached to the ends of the Maurer’s clefts that label with anti-HA/gold antibodies (**Figure 3B**, red arrows). Some labelling of the end of the Maurer’s cleft was also observed (**Figure 3B**, red arrows). These structures are distinct, smaller and not as electron dense, as the previously described electron dense vesicle (EDV) (Pachlatko et al., 2010) (**Figure 3B**, orange arrows). No gold labelling was observed in the antibody controls (**Supplementary Figure 3A**) nor following cKO of MAHRP2 (see section 4.2 below). This data shows that MAHRP2 locates at the tethers, Maurer’s clefts and small vesicles in NF54 parasites and that MAHRP2 labelled tether structures in NF54 have a different morphology to that observed in 3D7 background parasites.

### 3.2 Knockout of MAHRP2 leads to a loss of tethers and larger Maurer’s clefts

Previous work had suggested that MAHRP2 was refractory to deletion and thus may be an essential gene (Pachlatko et al., 2010). To test this, we induced cKO in the NF54-MAHRP2-HA parasites. Rapa or DMSO as a control was added from schizont stage (in the previous cycle) and harvested at the trophozoite stage in the following cycle (~30 hpi). PCR was used to confirm gene excision, as indicated by a shift in the size of the PCR product and a loss of MAHRP2-HA protein was confirmed by Western blot (**Figure 3D**; **Supplementary Figures 1A–D**). To examine if MAHRP2 influences parasite growth rate, we induced cKO and tracked development over 3 cycles. Deletion of MAHRP2 leads to a small but non-significant growth advantage, confirming it is not an essential gene (**Figure 3E**).

To determine if MAHRP2 is needed for tether formation we induced cKO of MAHRP2, harvested trophozoite stage parasites and labelled them with anti-HA followed by immuno-gold secondary antibody. As described above, tether structures were observed at the periphery of the clefts with additional free labelled tether structures within the cytoplasm in the controls (**Figure 3B**, red arrows). In contrast we did not observe any tethers structures or free tether structure in the cKO parasites consistent with MAHRP2 being required for their formation (**Figure 3C**). In addition to the loss of these structures, we observed an increase in the size of the Maurer’s clefts.

**Figure 4:**
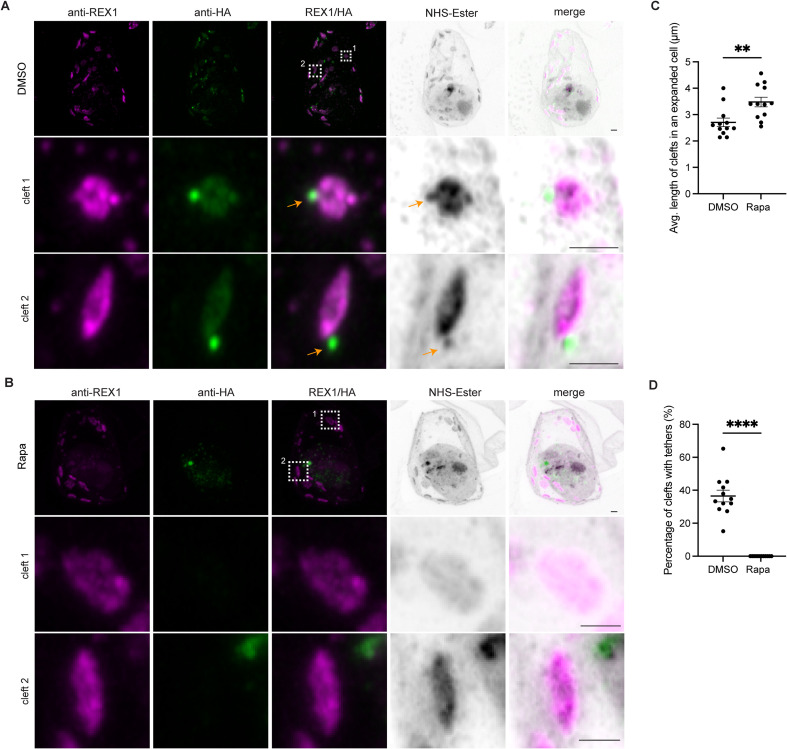
Deletion of MAHRP2 leads to longer Maurer’s clefts. **(A, B)** Expansion microscopy images of a DMSO and Rapa treated iRBC. Maurer’s clefts are labelled by anti-REX1 (magenta) and MAHRP2 labelled by anti-HA (green). NHS-Ester labels all proteins. Merged images of REX1/HA and REX1/HA/NHS-Ester are shown. Zoom images of maximum projections of z-stacks of single clefts are shown. In DMSO iRBC, MAHRP2 was shown co-staining the Maurer’s cleft and accumulating at distinct points (marked by orange arrows). Scale: 2 µm. **(C)** Average length of expanded Maurer’s clefts in DMSO (2.7 ± 0.6 µm) and Rapa (3.4 ± 0.6 µm) treated iRBCs were measured from ExM images, adjusted to the expansion factor and plotted. n = 3 biological repeats, 4 iRBC each, unpaired t-test p = 0.0047**. The mean values ± SD are plotted. **(D)** Graph showing the percentage of clefts with tethers. Each dot represents a cell. The percentage of Maurer’s clefts that had tethers is reported. n = 3 biological repeats, 12 cells analyzed in each group unpaired t-test p < 0.0001****. No tether structures were identified in the cKO parasites. The mean ± SEM are plotted.

Electron microscopy imaging was performed on unlabeled samples (**Supplementary Figure 3B**) and showed that the Maurer’s clefts were on average 212 nm wider (891 ± 234 nm) in the MAHRP2 cKO than in the control iRBCs (678 ± 285 nm) (**Figure 3F**). To confirm this enlarged cleft phenotype we next employed expansion microscopy (ExM). Airyscan imaging of expanded samples revealed bright HA staining of the tether at the cleft periphery in addition to weaker staining across the REX1 labelled Maurer’s clefts (**Figure 4A**). Counterstaining of the samples with an NHS-Ester dye shows brightly stained Maurer’s clefts and tether structures. Zoom images of 2 clefts in different orientations are shown (**Figure 4A**). Imaging of the cKO samples shows an absence of HA labelling and an absence of the NHS-Ester bright/MAHRP2 structures suggesting a loss of MAHRP2 labelled tether structures (**Figure 4B**). We further measured across the expanded clefts in both control and cKO iRBCs and observed that the cKO clefts (3.4 ± 0.6 µm) were ~700 nm wider compared to the control (2.7 ± 0.6 µm) confirming our EM observations (**Figure 4C**). We next evaluated the number of clefts that contained NHS-Ester bright puncta associated with the Maurer’s clefts. In controls we see on average 36.5% of the Maurer’s clefts in each cell have identifiable tethers (**Figure 4D**). No tethers were observed following cKO of MAHRP2 (**Figure 4D**). Quantification of the number of Maurer’s clefts using REX1 labelling in standard IFA showed no significant difference in numbers between the cKO and DMSO controls (**Supplementary Figures 4A, B**). Given these differences we then checked the abundance of REX1, SBP1 or MAHRP1 protein expression following cKO of MAHRP2 which showed no change in protein expression despite the changes to cleft structure (**Supplementary Figure 4C**).

### 3.3 Deletion of MAHRP2 leads to increased Maurer’s cleft movement

**Figure 5:**
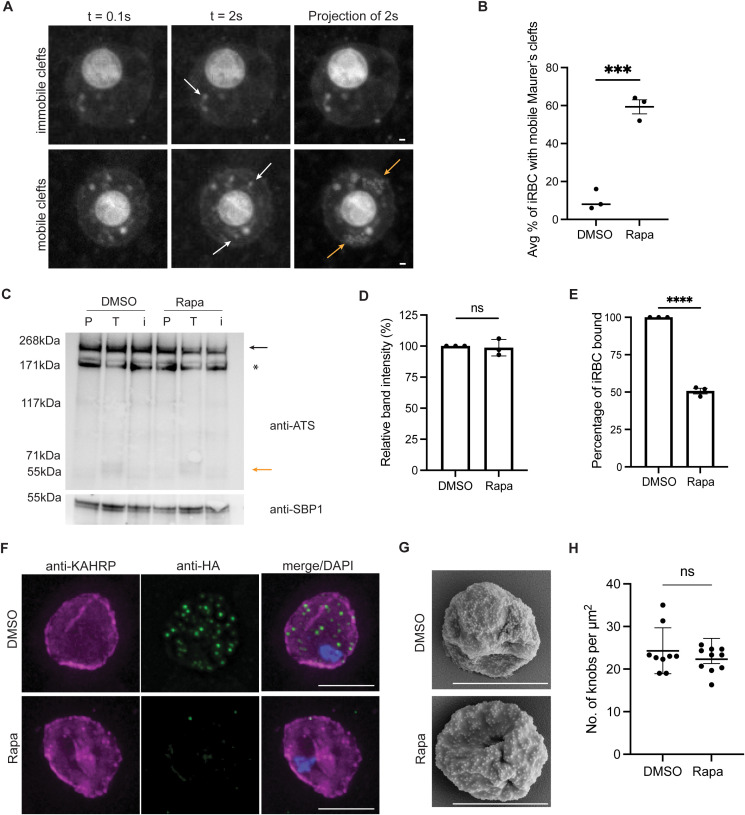
Deletion of MAHRP2 results in reduced cytoadhesion. **(A)** Images of BODIPY-ceramide labelled cells showing a single image frame at t = 0.1s and 2s, and a projection image of 20 frames captured over 2 seconds highlighting mobile and immobile clefts. The yellow arrows point to mobile clefts which are observed as a “cloud”. Scale: 500 nm. **(B)** Scatter plot showing the percentage of Maurer’s cleft in motion in DMSO and Rapa treated iRBCs. The mean values ± SEM are plotted, n = 3 biological repeats, 50 cells were observed in each experiment. 49 ± 3% increase in mobile clefts were observed in Rapa treated cells compared to the DMSO cells (unpaired t-test, p = 0.0005***). **(C)** Trypsin cleavage assay of DMSO and Rapa treated MAHRP2-HA iRBCs. P: PBS control, T: trypsin treated, i: Trypsin + trypsin inhibitor. The yellow arrow points to the 55kDa ATS cleavage band. The full-length *Pf*EMP1 band is marked by a black arrow and cross-reaction band with RBC spectrin is marked by an asterisk. Anti-SBP1 was used as a loading control. **(D)** Graph showing densitometric quantification of anti-ATS bands normalized to the anti-SBP1 loading control. n = 3 biological replicated, paired t-test p =0.7618ns. **(E)** Binding of infected RBC to CD36 ligand under flow conditions. DMSO and Rapa treated MAHRP2 infected RBCs were analysed. Unpaired t test p <0.0001****, n= 3 biological replicates, experiment performed in triplicate. The mean values ± SEM are plotted. **(F)** Immunofluorescence microscopy of DMSO and Rapa treated infected RBCs probed with anti-HA (green) and anti-KAHRP (magenta). A merge of the 2 channels and the DNA label (DAPI) are shown. Scale: 5 µm. **(G)** External scanning electron microscopy (SEM) showing knob morphology of DMSO and Rapa treated infected RBCs. Scale: 5 µm. **(H)** Graph showing number of knobs per square micron of DMSO (n= 9 cells) and Rapa (n= 10 cells) treated iRBCs. p= 0.342ns, Welch’s t test.

The tethers have been previously hypothesized to play a role in the immobilization of Maurer’s clefts to the RBC membrane skeleton (McMillan et al., 2013). To examine if the deletion of MAHRP2 and the loss of tethers impacts cleft immobilization we performed live cell fluorescence microscopy on synchronized trophozoites (24–28 hpi), labelled with the membrane dye BODIPY-TR-ceramide. BODIPY labels all membranes within the iRBC including the Maurer’s clefts which can be seen as puncta in the RBC cytoplasm (**Figure 5A**, white arrow). To visualize cleft movement a 2 s video consisting of 20 images was acquired and a projection image generated. Immobile clefts are observed as single puncta (white arrow), while mobile clefts are observed as a “cloud” (**Figure 5A**, yellow arrows). If mobile clefts are observed this cell is classified as being mobile, quantification of individual clefts is not conducted. In the DMSO control only 10% of the cells had mobile Maurer’s clefts, showing that the clefts have immobilized in the majority if cells in this population. In contrast 59% of the iRBCs in the Rapa treated group exhibited mobile Maurer’s clefts (**Figures 5A, B**). This data suggests that MAHRP2 and tethers play a role in cleft immobilization but are not the only mechanism at play.

### 3.4 Knockout of MAHRP2 reduces iRBC binding to CD36

It has been previously suggested that the tethering of the Maurer’s cleft to the RBC membrane is required for efficient *Pf*EMP1 trafficking (Cyrklaff et al., 2011). To understand the role of tethers in *Pf*EMP1 transport, a trypsin cleavage assay was performed to qualitatively assess if cKO of MAHRP2 alters *Pf*EMP1 display at the iRBC membrane. Whole intact parasite infected RBCs were treated with trypsin (T) to cleave the extracellular domains of *Pf*EMP1, leaving behind the conserved cytoskeleton bound acidic terminal segment (ATS) of *Pf*EMP1. Controls for this experiment include PBS treated iRBCs (P) and iRBCs incubated with trypsin inhibitor prior to trypsin treatment (i). The ATS region was then extracted from the cell membrane and analyzed by Western blotting with antibodies to the ATS region. A band of ~55 kDa corresponding to the ATS region of the NF54 strain was observed in the trypsin treated samples of both the control and cKO showing that a population of *Pf*EMP1 is delivered to the iRBC membrane in both control and MAHRP2 cKO iRBCs (**Figure 5C**). Quantification of the surfaced exposed ATS bands show that there is no significant difference in the total *Pf*EMP1 reaching the RBC surface (**Figure 5D**).

We next assessed the ability of the MAHRP2 cKO cells to bind to the human endothelial ligand CD36, under physiological flow conditions. Trophozoite iRBCs (24–32 hpi) were passed through a narrow chamber coated with CD36 and the number of iRBCs bound in 10 randomly chosen fields was recorded. We observed a 49% reduction in iRBC binding to CD36 following cKO of MAHRP2 when compared to controls (**Figure 5E**). Given the trypsin cleavage results showing no change in *Pf*EMP1 surface exposure the loss of adhesion may be due to a defect in the knobs.

The knob structure assembled at the iRBC membrane plays an important role in the display of *Pf*EMP1 and in enhancing the adherence of iRBC to endothelial cell walls under physiological flow conditions (Rug et al., 2006). IFAs with antibodies against the Knob Associated Histidine Rich Protein (KAHRP) showed no change in the fluorescence pattern following knockout of MAHRP2 (**Figure 5F**). To further check if conditional knockout of MAHRP2 influences knob density or morphology, knockdown parasites were analyzed by scanning electron microscopy (SEM). There was no visible difference in the appearance of knobs following knockout when compared to untreated controls (**Figure 5G**). There was also no significant difference in the number of knobs on the surface of the control (24 ± 5 knobs/µm^2^) and cKO (22 ± 3 knobs/µm^2^) iRBC (**Figure 5H**; **Supplementary Figure 4D**). This suggested that the loss of adherence phenotype is most likely due to the abnormal presentation of *Pf*EMP1 at the surface and not altered knob morphology.

### 3.5 Knockout of MAHRP2 results in decreased abundance of many exported proteins

**Figure 6:**
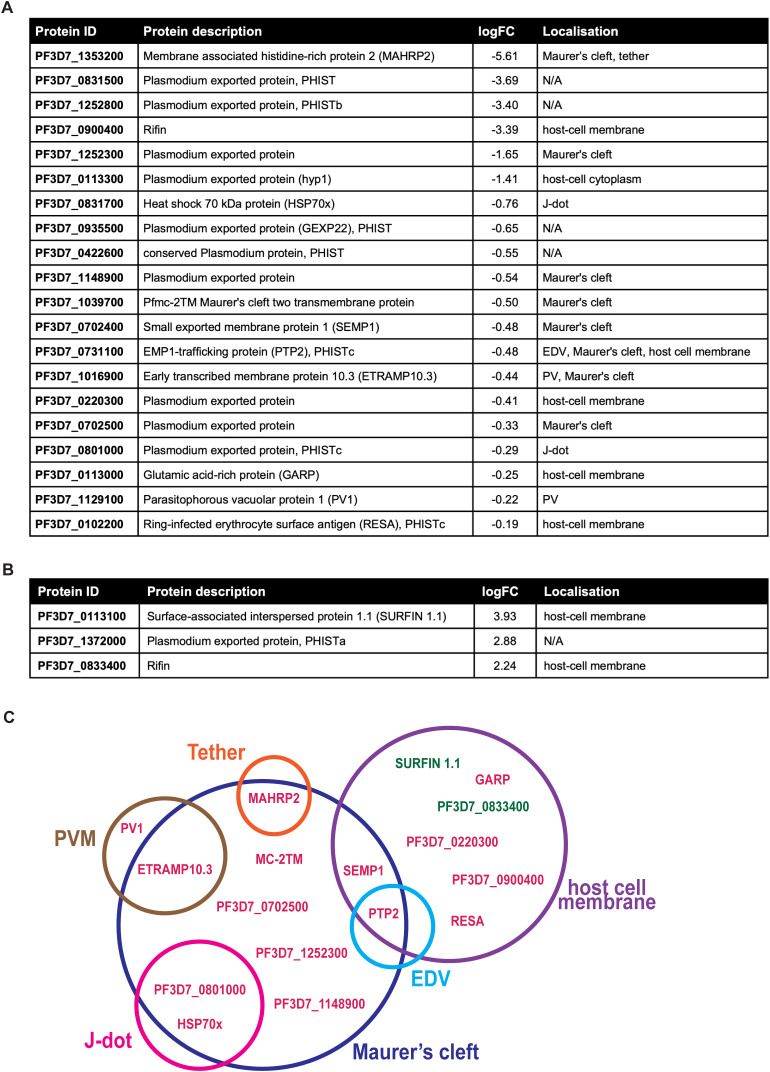
Whole cell proteomics analysis of MAHRP2 knockout cells. Collated list of previously described exported proteins identified in the global proteomics analysis. **(A)** Proteins that are significantly less abundant and **(B)** more abundant in MAHRP2 cKO compared to control cells. Protein ID, protein description, log fold-change and localization are reported. N/A = not available. **(C)** Venn diagram of the localization of exported proteins that significantly changed in the absence of MAHRP2. Sets – Maurer’s cleft (dark blue), host cell membrane (purple), tether (orange), parasitophorous vacuole membrane (PVM) (brown), J-dot (magenta) and electron dense vesicles (EDV, light blue). Significantly less abundant proteins are in red and significantly more abundant proteins are in green.

To investigate if deletion of *mahrp2* leads to global changes in exported proteins we conducted label free whole cell quantitative proteomics. In this analysis trophozoite stage parasites were examined and the proteins differentially expressed in the knockout identified. The protein hits were further filtered to focus on those with GO terms relating to exported proteins and excluding those with “nuclear”, “mitochondrial” or “apicoplast” terms. This collated list of proteins can be found in **Figure 6A**, and the full list of all differentially expressed proteins can be found in the **Supplementary Table 1**.

As expected MAHRP2 was the most reduced protein with a forty-nine-fold reduction (logFC = -5.61) compared to the control. No changes were observed in REX1, SBP1 or MAHRP1, consistent with our Western results (**Supplementary Figure 4C**). However, significant decreases were observed in the MAHRP2 cKO, for some exported parasite proteins known to be associated with the Maurer’s cleft, parasitophorous vacuole (PV), host cell membrane, J-dots and EDVs. Other significant hits include 27 uncharacterized proteins with some associating to the GO Term “integral component of membrane” (**Supplementary Figure 5**). Several Maurer’s cleft proteins were identified as being reduced including the known MAHRP2 interacting proteins SEMP1 and PF3D7_0702500 (Dietz et al., 2014), the *Plasmodium falciparum* Maurer’s cleft two transmembrane protein (*Pf*mc-2TM) PF3D7_1039700 and putative Maurer’s cleft proteins PF3D7_1252300 and PF3D7_1148900 (Heiber et al., 2013). The PV proteins Early Transcribed Membrane Protein 10.3 (ETRAMP10.3) and Parasitophorous Vacuolar protein 1 (PV1) which has previously been linked to *Pf*EMP1 trafficking (Nyalwidhe and Lingelbach, 2006; Mackellar et al., 2010; Batinovic et al., 2017) were also slightly reduced. Other proteins that have previously been linked to *Pf*EMP1 trafficking were also identified as being down in the cKO including the electron dense vesicle protein *Pf*EMP1-trafficking protein 2 (PTP2) (McHugh et al., 2020) and the J-dot chaperone protein, exported heat-shock protein 70 (HSP70x) (Wilde et al., 2019). Another J-dot associated protein PF3D7_0801000 with an unknown function (Parkyn Schneider et al., 2017) along with 7 members of the PHIST/PRESAN family of proteins including PF3D7_0831500 (PHISTb), PF3D7_1252800 (PHISTb), PF3D7_0935500 (GEXP22), PF3D7_0422600 and the Ring-infected Erythrocyte Surface Antigen (RESA) were found to be reduced. An uncharacterized exported protein PF3D7_1148900 was also found to be reduced.

Very few proteins were identified as being significantly upregulated in the MAHRP2 cKO when compared to the control. The enriched exported proteins included the surface-associated interspersed protein 1.1 (SURFIN 1.1), a RIFIN (PF3D7_0833400) and a PHISTa (PF3D7_1372000) protein (**Figure 6B**). Other significant hits included two uncharacterized proteins - PF3D7_1448100 and PF3D7_0820300 (**Supplementary Figure 5**).

Collectively these data point towards a significant global impact on trafficking in the absence of MAHRP2 and highlights the interconnectivity of the exomembrane system. **Figure 6C** highlights the interconnectivity of the dysregulated proteins relative to each other and the structures they locate at.

### 3.6 Knockout of MAHRP2 leads to PTP2 accumulation at the clefts.

Previous studies investigating *Pf*EMP1 trafficking from the Maurer’s clefts to the iRBC surface have suggested a role for J-dot chaperone complexes and Maurer’s cleft derived EDVs in *Pf*EMP1 trafficking (Charnaud et al., 2017; Carmo et al., 2022). Considering this literature and the disruption of J-dot and EDV proteins in our whole cell proteomics data we examine key components of these structures. HSP70x is the sole exported HSP70 protein which interacts with exported HSP40 co-chaperones to form J-dot complexes (Charnaud et al., 2017; Behl et al., 2019), and PTP2 is an EDV protein. PTP2 containing vesicles have been reported to bud off the Maurer’s clefts as the parasite matures, and deletion of PTP2 leads to a dramatic reduction in surface exposed *Pf*EMP1 (Maier et al., 2008; Regev-Rudzki et al., 2013).

Firstly, we examined the localization of the J-dot protein HSP70x in 24–28 hpi control and cKO cells by performing an IFA anti-REX1 and anti-HSP70x antibodies. We see no change in the location of the HSP70x following cKO of MAHRP 2 compared to controls, suggesting that its deletion does not affect J-dot biology (**Supplementary Figure 6A**).

**Figure 7:**
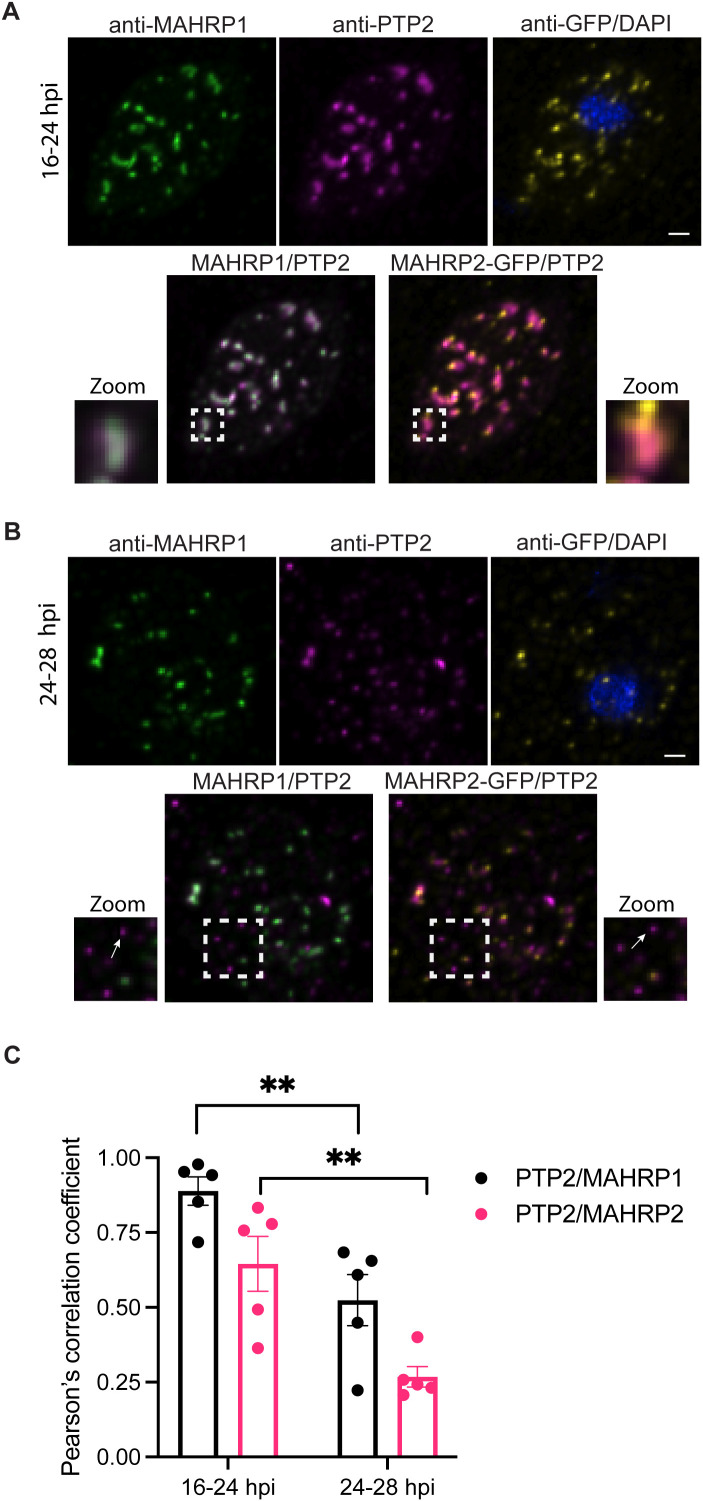
PTP2 vesiculates from the Maurer’s clefts at 24–28 hpi. **(A, B)** Super-resolution microscopy of MAHRP2-GFP cells triple-labelled using anti-GFP (yellow), anti-PTP2 (magenta), anti-MAHRP1 (green) and the DNA label (DAPI; blue) are shown. A merge of the MAHRP1/PTP2 channels and MAHRP2-GFP/PTP2 are shown. **(A)** Parasites of age 16–24 hpi were imaged. Zoomed images of a single Maurer’s cleft showing that PTP2 co-locates with MAHRP1 in the inner Maurer’s cleft region and only partially co-locates with MAHRP2 in the cleft periphery. Scale bar – 500 nm. **(B)** Parasites of age 24–28 hpi were imaged. Zoomed images show magenta PTP2 puncta in the cytoplasm independent of Maurer’s clefts. **(C)** Colocation of PTP2 with MAHRP1 and MAHRP2 at the two timepoints was measured using Pearson’s correlation coefficient and plotted. PTP2 co-locates with MAHRP1 significantly more at 16–24 hpi (0.88 ± 0.12) than 24–28 hpi (0.52 ± 0.19) (unpaired t-test, p=0.0058**, n=5). PTP2 co-locates with MAHRP2 significantly more at 16–24 hpi (0.64 ± 0.20) than 24–28 hpi (0.27 ± 0.08) (unpaired t-test, p=0.0048**, n=5). The mean values ± SEM are plotted.

Next, we examined the location of PTP2 within the Maurer’s clefts (Carmo et al., 2022) during early (16–24 hpi) and late (24–28 hpi) trafficking windows before and after vesicle formation. To do this we performed super-resolution microscopy on a MAHRP2-GFP cell line labelled with anti-MAHRP1 and anti-PTP2 antibodies (McMillan et al., 2013) (**Figures 7A, B**). In early trophozoite stages (16–24 hpi) before PTP2 vesicles are formed we see a high level of PTP2 co-location with MAHRP1 in the inner compartment of the Maurer’s cleft (Pearson’s coefficient (r) = 0.89) (**Figures 7A, C**). In addition to this we see a modest level of PTP2 overlapping with the MAHRP2 population present at the periphery of the clefts and no labelling of PTP2 on the tether itself (r = 0.52) (**Figures 7A, C**). At the later timepoint (24–28 hpi) there is a significant reduction in the overlap of PTP2 with MAHRP1 (r = 0.52) and MAHRP2 (r= 0.27) which corresponds with an increase in PTP2 labelled puncta (vesicles) observed in the RBC cytoplasm (**Figure 7B**, (white arrow)). Additional examples are shown in **Supplementary Figures 6B, C**.

**Figure 8:**
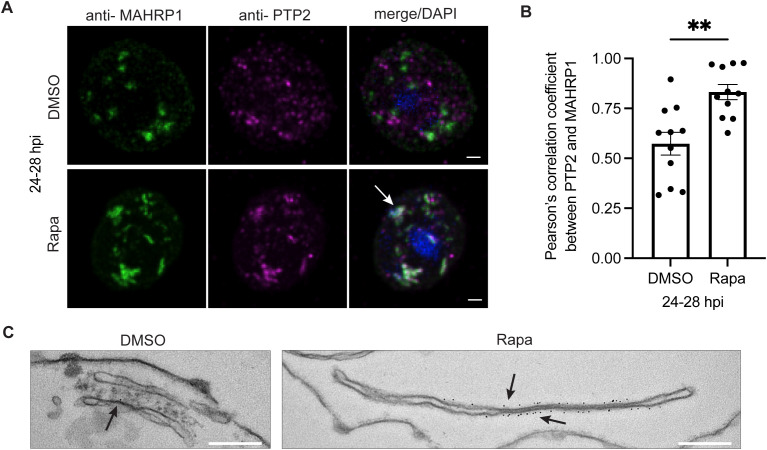
Effect of MAHRP2 knockout on PTP2 vesiculation. **(A)** Super-resolution microscopy of DMSO and Rapa treated MAHRP2-HA cells (24–28 hpi) probed with anti-PTP2 (magenta) and anti-MAHRP1 (green). A merge of the 2 channels and DAPI are shown. Scale: 500 nm. **(B)** Change in localization of PTP2 in MAHRP2 cKO cells were measured using Pearson’s coefficient. Co-location coefficient of MAHRP1/PTP2 was significantly more in the KO (0.83 ± 0.12) than in the controls (0.57 ± 0.19); (unpaired t-test, p=0.0012**, n=11). The mean values ± SEM are plotted. **(C)** Immuno-EM of DMSO and Rapa treated parasites with anti-PTP2 primary labelling and gold bead secondary labelling. MAHRP2 cKO showing abundant labelling of PTP2 (black arrows) on the periphery of a long Maurer’s cleft. Scale bar – 200 nm.

Having established the localization pattern and timing of vesiculation, we examined if the location of PTP2 was altered following MAHRP2 deletion. Immunofluorescence microscopy at 16–24 hpi shows PTP2 at the Maurer’s clefts in both control and cKO cells (**Supplementary Figure 6C**). As shown previously, control cells at 24–28 hpi show small PTP2 labelled vesicle structures within the cytoplasm of the iRBC, with some co-location at the Maurer’s clefts (**Figure 8A**, DMSO). In contrast following cKO of MAHRP2 the fluorescence profile of PTP2 shows an increase in the co-location of MAHRP1 and PTP2 in the cKO cells (Pearson’s coefficient, 0.77) when compared to controls (0.52) (**Figure 8B**).

To further examine this phenotype, we employed immunogold labelling of PTP2 in DMSO and Rapa treated iRBCs at 24–28 hpi. In control cells we see limited PTP2 gold-labelling consistent with PTP2 being largely absent from Maurer’s clefts later in development (**Figure 8C**). In contrast we see a significant amount of PTP2-gold labelling across the central part of the Maurer’s clefts in the cKO parasites consistent with our immunofluorescence observations (**Figure 8C**). Additional examples are shown in **Supplementary Figure 7B**.

## 4 Discussion

The “tether” structure was first identified in electron micrographs and was described as a tube-like or electron dense structure connecting the Maurer’s clefts to the RBC membrane (Hanssen et al., 2008). The function of the tether remained unknown with studies suggesting that it may function in *Pf*EMP1 trafficking through the direct tethering of the clefts to the RBC membrane skeleton (Pachlatko et al., 2010). The only known tether protein is MAHRP2, which is expressed early in development in structures that were shown, using conventional fluorescence microscopy, to lie adjacent to the Maurer’s clefts (Pachlatko et al., 2010). In this study we show that MAHRP2 labelled Maurer’s clefts and tether structures, and the appearance of Maurer’s clefts are different in NF54 when compared to 3D7 parasites. Our immunofluorescence and immuno-EM studies show that MAHRP2 labels rounder fuzzier structures at the periphery of the Maurer’s clefts in NF54, which differs from the tube-like structure describe in 3D7 (Hanssen et al., 2008). These immuno-EM studies also identify free tether structures that label with MAHRP2 within the iRBC cytoplasm in mature trophozoites. Using Airyscan super resolution microscopy and expansion microscopy we show that MAHRP2 locates at the Maurer’s cleft periphery in addition to the tether itself where it locates with SBP1 and REX1 proteins known to locate to the outer compartment of the Maurer’s cleft (McHugh et al., 2020). Confirmation that these structures are likely tethers is provided by our immuno-EM and expansion microscopy data showing that following cKO of MAHRP2 we see a loss of both the free and Maurer’s cleft associated tether structures. Concurrent with the loss of these structures we also observe a significance increase in the size of the Maurer’s clefts following cKO and a loss of the MAHRP2 vesicles from the cytoplasm. This raises the possibility that the tether structures and vesicle structures may originate from the Maurer’s’ clefts. The origins of these structures could be an interesting avenue to peruse in future studies.

Published co-immunoprecipitation experiments using REX1 identified MAHRP2, an uncharacterized protein PF3D7_0702500 and the *Pf*EMP1 trafficking protein- 6 (PTP6) as interacting partners (Maier et al., 2008; McHugh et al., 2020). Both REX1 and PTP6 have been linked to *Pf*EMP1 trafficking and locate at the cleft periphery. The co-location of MAHRP2 with these important *Pf*EMP1 trafficking proteins may explain how the changes to cleft structure and the loss of the tethers from the periphery in the MAHRP2 cKO results in a significant reduction in cytoadhesion. The reduced ability of the MAHRP2 cKO to adhere may be due to a *Pf*EMP1 display defect. Similar findings have been previously reported where the amount of *Pf*EMP1 at the surface is not affected but adhesion is reduced (Charnaud et al., 2017). One interpretation of these data is that these proteins are important for the trafficking of additional proteins that are required for the loading of *Pf*EMP1 into the RBC membrane and their subsequent correct presentation at the knobs. Further research is required in this space to understand how Maurer’s cleft, vesicle and chaperone proteins interact and work together to remodel the host cell membrane allowing for efficient display of *Pf*EMP1.

Previous studies have shown that the Maurer’s clefts are highly mobile in the RBC cytoplasm during early development becoming immobilized under the RBC membrane later in development. Here we show that cKO of MAHRP2 results in a significant increase in the number of iRBCs (~50%) that still exhibit mobile Maurer’s clefts suggesting that MAHRP2 and tethers may contribute to cleft immobilization. This points towards other additional factors that may contribute to cleft immobilization to this point, a recent study investigating the resident Maurer’s cleft proteins PIESP2 and *Pf*332 have shown that deletion of these genes also effects tethering and cleft architecture (Blancke Soares et al., 2025). Collectively this points towards multiple proteins controlling Maurer’s cleft tethering to the RBC membrane and it may be that cleft immobilization is simply a function of reduced space in the iRBC cytoplasm forcing the clefts into closer proximity to the RBC membrane. Nonetheless it is unclear the question of what controls cleft immobilization and how critical this is to trafficking remains unresolved.

It is clear that the protein trafficking networks functioning during host cell remodeling are complex (McHugh et al., 2020; Jonsdottir et al., 2021). In an effort to take an unbiased look at what proteins were changing following cKO of MAHRP2 we conduced global label free proteomics of MAHRP2 cKO and control iRBCs. In this data we see a reduction in protein expression for markers of the chaperone containing J-dot structures, Maurer’s cleft proteins and the EDV protein PTP2. This data supports previous studies that have shown that these structures are important for *Pf*EMP1 trafficking and highlights the interconnectivity of the protein trafficking pathways that function in each of the different structures that comprise the exomembrane system. The complexity of this system reflects the importance of host cell remodeling and virulence protein trafficking has on parasite survival within the hosts circulation. Interestingly, in addition to the reduction of exomembrane components we also observe a reduction in multiple proteins that have been shown to locate at the RBC membrane, these include multiple uncharacterized members of the PHIST family of proteins including one of which has a Mature Parasite-infected Erythrocyte Surface Antigen (MESA) erythrocyte cytoskeleton-binding (MEC) motif (PF3D7_1252800) (Kilili and LaCount, 2011). These proteins could be potential membrane binders helping in the docking of the Maurer’s clefts to the RBC membrane. Future work to profile the exomembrane compartments in a more targeted way, through proximity tagging approaches or cellular fractionation may help identify more subtle changes that have been missed in our global proteomics approach.

Previous work has shown that EDVs contain *Pf*EMP1 and have been observed attached to the Maurer’s clefts and tethers, free in the RBC cytoplasm and docked at the RBC membrane. The exported protein PTP2 is a marker of EDVs (Regev-Rudzki et al., 2013). In this work we show a change in PTP2 location following deletion of MAHRP2. Our data shows that PTP2 initially locates at the Maurer’s clefts prior to being loaded into the EDVs. Free EDVs in the cytoplasm are difficult to observe by thin section EM, but the movement of PTP2 from the clefts to free EDVs in the cytoplasm can be observed by fluorescence imaging. In this work we see retention of PTP2 at the Maurer’s clefts following deletion of MAHRP2. The inability to efficiently form EDVs may underly the loss of adhesion observed in our studies. Our previous work and the work shown here show considerable compartmentalization of protein complexes within the Maurer’s clefts, which appear to function in the trafficking of *Pf*EMP1 and other virulence related cargoes. It is hypothesized that the location and composition of the complexes are important for *Pf*EMP1 trafficking into the central parts of the clefts and *Pf*EMP1 trafficking out of the cleft periphery (McHugh et al., 2020). Another factor contributing to the trafficking of proteins is the architecture of the cleft itself and the formation and release of vesicles. Knockout studies of PTP1 and REX1 lead to shorter clefts and cleft stacking respectively, while a knockout of PTP7 leads to the mass accumulation of vesicles at the cleft. In each of these cases these physical changes contribute to significant defects in trafficking (McHugh et al., 2015; McHugh et al., 2020; Carmo et al., 2022). This provides some support that the physical changes in cleft architecture and vesicle formation in the absence of MAHRP2 may be contributing to the phenotypes observed and the reduction in the parasite's ability to cytoadhere.

## Data Availability

The datasets presented in this study can be found in online repositories. The names of the repository/repositories and accession number(s) can be found in the article/**supplementary Material**.

## Supplementary material

Supplementary Figure 1 Characterisation of the MAHRP2-HA parasite line **(A)** Schematic illustrating the genomic locus of *mahrp2* pre and post integration, and post addition of Rapa. HR 1 & 2 = Homology region 1 & 2; recodon (dark grey) = re-codonised 3’ *mahrp2* sequence; HA (orange) = 3x Haemagglutinin tag; *loxP* sites are shown in green. *glmS* (yellow) = Glucosamine riboswitch; WR (brown) = human dihydrofolate reductase drug cassette. Primer locations are marked with arrows and have been designated with letter A through D. **(B)** PCR validation confirming integration of the plasmid into the *mahrp2* locus and diCRE mediated excision. NF54 parent gDNA was used as a control. **(C)** PCR validation confirming deletion of the *mahrp2* locus following Rapa treatment. DMSO treated iRBC were used as a control. Gene excision is confirmed by the shift in size of PCR product. **(D)** List of the primers used to generate the MAHRP2-HA conditional knockout cell line and PCR validations.

Supplementary Figure 2 Super resolution imaging and quantification of MAHRP2-HA localization. **(A–C)** Additional example images of MAHRP2 location at the Maurer’s clefts. MAHRP2-HA (green) co-stained with anti-REX1 (A, magenta), anti-SBP1 (**B**, magenta) and anti-MAHRP1 (**C**, magenta). **(D)** MAHRP2-GFP (green) and anti-REX1 (magenta). Zoomed images are of single Maurer’s clefts showing accumulation of MAHRP2 at distinct points (yellow arrows) on the outer compartment are shown. Scale: 500 nm. **(E)** Pearson’s correlation coefficient analysis of MAHRP2 colocation with REX1, SBP1 and MAHRP1. n = 6 cells. Unpaired t-test was performed to confirm a significant increase in colocation between MAHRP2/REX1 and MAHRP2/MAHRP1, p<0.0001**** and MAHRP2/SBP1 and MAHRP2/MAHRP1 p= 0.0013**); MAHRP2/REX1 and MAHRP2/SBP1 co-location coefficient are not significantly different (unpaired t-test, p = 0.0611 ns). **(F)** Table showing the mean Pearson’s correlation coefficient values of REX1/SBP1/MAHRP1 versus MAHRP2-HA staining.

Supplementary Figure 3 The effect of MAHRP2 cKO on Maurer’s cleft morphology. **(A)** Negative control for immuno-EM. NF54-MAHRP2-HA labelled with mouse anti-beta tubulin primary raised in the same animal showing no labelling confirming absence of non-specific binding of the gold secondary antibody. Possible MAHRP2 vesicles are indicated with red arrows. Maurer’s clefts – white arrows. Scale: 200 nm. **(B)** Additional images for the unlabeled thin-section TEM of DMSO and Rapa treated iRBC. Maurer’s clefts are shown in both DMSO and Rapa treated iRBCs. Tethers (indicated with red arrow) were observed in DMSO treated iRBCs only. Scale: 200 nm. **(C)** Additional examples of expansion microscopy images of a DMSO and Rapa treated iRBC. Maurer’s clefts are labelled by anti-REX1 (magenta) and MAHRP2 labelled by anti-HA (green). NHS-Ester labels all proteins. Merged images of REX1/HA and REX1/HA/NHS-Ester are shown. Zoom images of maximum projections of z-stacks of single clefts are shown.

Supplementary Figure 4 The effect of MAHRP2 cKO on Maurer’s cleft proteins and knobs **(A)** Immunofluorescence imaging of DMSO and Rapa treated MAHRP2-HA cells probed with anti-SBP1 and anti-MAHRP1 to examine the Maurer’s clefts. Scale: 500 nm. **(B)** Bar graph showing number of Maurer’s cleft in the DMSO and Rapa treated MAHRP2-HA cells. p = 0.60 ns, n= 25 cells from 3 biological replicates. **(C)** Full uncut Western blots (of **Figure 1D**) at 3s exposure and 12s exposure showing equal loading of saponin pellets of MAHRP2-HA cells treated with DMSO or Rapa. Loss of anti-HA signal in Rapa treated sample confirms protein knockout. Anti-ERC was used as a loading control. Anti-REX1, anti-SBP1 and anti-MAHRP1 antibodies were used to examine Maurer’s cleft protein expression; no apparent difference in between the DMSO and Rapa samples. **(D)** Additional images of external SEM of control and cKO iRBC. Scale: 4 µm.

Supplementary Figure 5 List of uncharacterized *Plasmodium falciparum* proteins identified to be significantly depleted or enriched in the MAHRP2 cKO cells. log(Fold Change) and GO Terms where available are shown, N/A - not available.

Supplementary Figure 6 Effect of MAHRP2 cKO on HSP70x and triple-labelling of MAHRP2-GFP **(A)** Super-resolution microscopy of DMSO and Rapa treated MAHRP2-HA cells (24–28 hpi) probed with anti-REX1 (magenta) and anti-HSP70x (green). A merge of the 2 channels and DAPI are shown. Scale: 500 nm. **(B, C)** Additional images for MAHRP2-GFP cells triple-labelled using anti-GFP (yellow), anti-PTP2 (magenta), anti-MAHRP1 (green) and the DNA label (DAPI; blue) are shown. A merge of the MAHRP1/PTP2 channels and MAHRP2-GFP/PTP2 are shown. **(B)** Parasites of age 16–24 hpi were imaged. Zoomed images of a single Maurer’s cleft showing that PTP2 co-locates with MAHRP1 in the inner Maurer’s cleft region and only partially co-locates with MAHRP2 in the cleft periphery. Scale bar – 500 nm. **(C)** Parasites of age 24–28 hpi were imaged. Zoomed images show magenta PTP2 puncta in the cytoplasm independent of Maurer’s clefts.

Supplementary Figure 7 Effect of MAHRP2 cKO on PTP2 **(A)** Super-resolution microscopy of 16–24 hpi DMSO and Rapa treated MAHRP2-HA cells labelled using anti-MAHRP1 (green), anti-PTP2 (magenta) and the DNA label (DAPI; blue) are shown. A merge of the 2 channels and DAPI are shown. Scale: 500 nm. PTP2 is observed to be located at the Maurer’s cleft in both DMSO and Rapa treated iRBC at this age window. **(B)** Additional images of DMSO and Rapa treated parasites with anti-PTP2 primary labelling and gold bead secondary labelling. MAHRP2 cKO showing abundant labelling of PTP2 (black arrows) on the periphery of Maurer’s clefts. Scale bar – 500 nm.
