# Supplementary figures and images for: MAHRP2 is required for tether formation and cytoadherence in Plasmodium falciparum infected red blood cells
Source: Front Cell Infect Microbiol. 2026 May 29;16:1675134. doi: 10.3389/fcimb.2026.1675134 (PMC13259744; doi:10.3389/fcimb.2026.1675134)

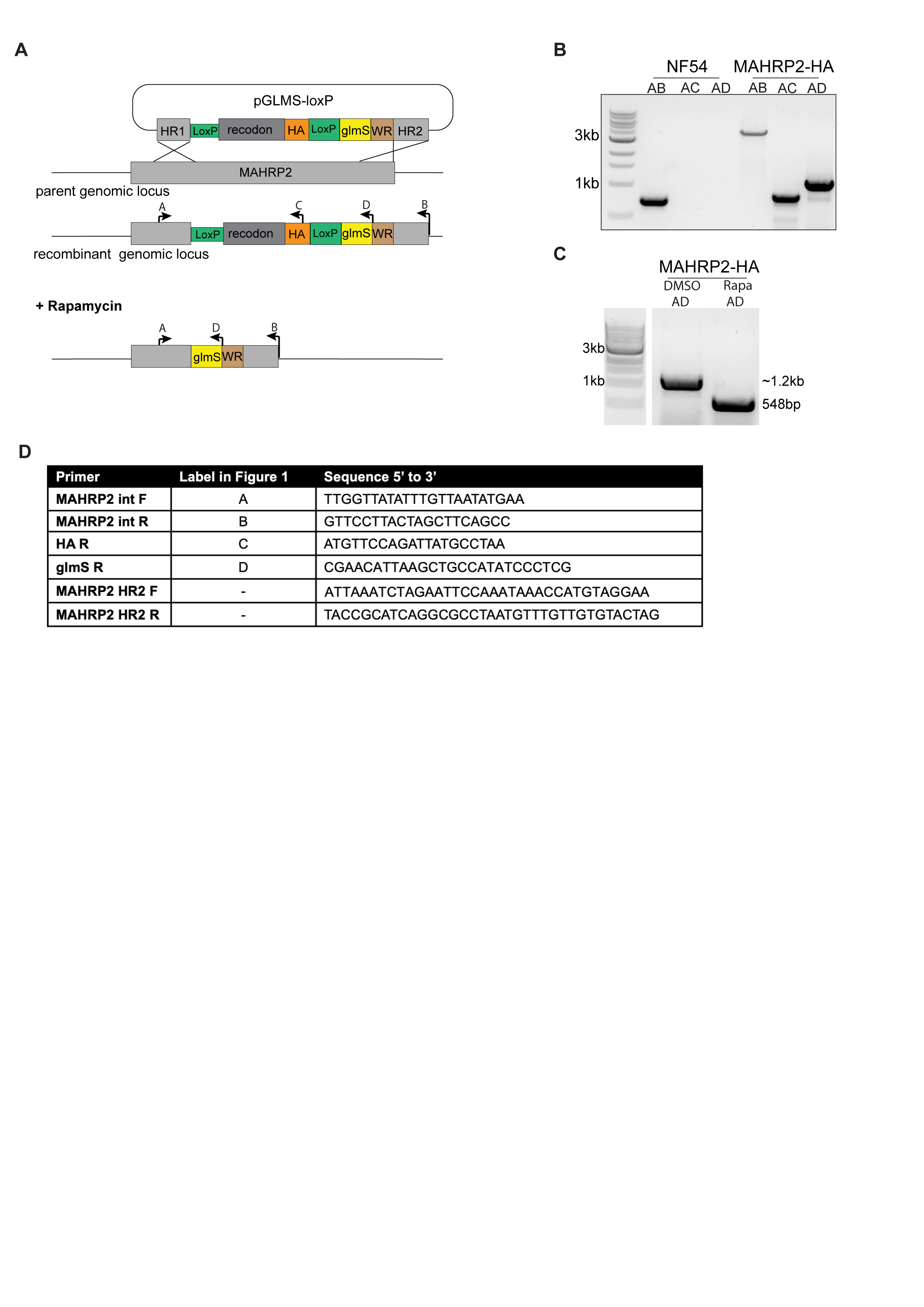

Supplement: Supplementary Figure 1 — Characterisation of the MAHRP2-HA parasite line (A) Schematic illustrating the genomic locus of mahrp2 pre and post integration, and post addition of Rapa. HR 1 & 2 = Homology region 1 & 2; recodon (dark grey) = re-codonised 3’ mahrp2 sequence; HA (orange) = 3x Haemagglutinin tag; loxP sites are shown in green. glmS (yellow) = Glucosamine riboswitch; WR (brown) = human dihydrofolate reductase drug cassette. Primer locations are marked with arrows and have been designated with letter A through D. (B) PCR validation confirming integration of the plasmid into the mahrp2 locus and diCRE mediated excision. NF54 parent gDNA was used as a control. (C) PCR validation confirming deletion of the mahrp2 locus following Rapa treatment. DMSO treated iRBC were used as a control. Gene excision is confirmed by the shift in size of PCR product. (D) List of the primers used to generate the MAHRP2-HA conditional knockout cell line and PCR validations. [file Image1.tif]

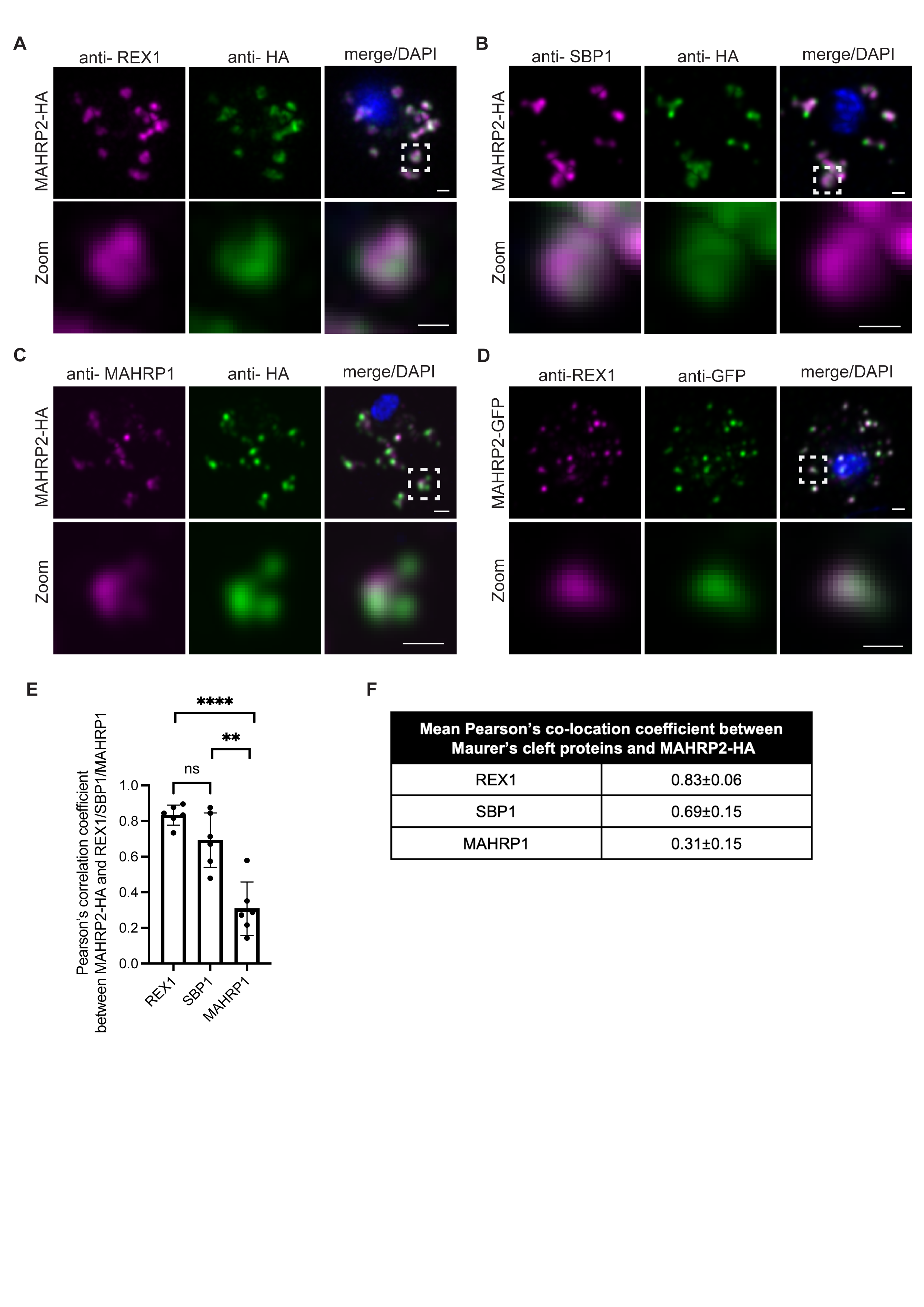

Supplement: Supplementary Figure 2 — Super resolution imaging and quantification of MAHRP2-HA localization. (A–C) Additional example images of MAHRP2 location at the Maurer’s clefts. MAHRP2-HA (green) co-stained with anti-REX1 (A, magenta), anti-SBP1 (B, magenta) and anti-MAHRP1 (C, magenta). (D) MAHRP2-GFP (green) and anti-REX1 (magenta). Zoomed images are of single Maurer’s clefts showing accumulation of MAHRP2 at distinct points (yellow arrows) on the outer compartment are shown. Scale: 500 nm. (E) Pearson’s correlation coefficient analysis of MAHRP2 colocation with REX1, SBP1 and MAHRP1. n = 6 cells. Unpaired t-test was performed to confirm a significant increase in colocation between MAHRP2/REX1 and MAHRP2/MAHRP1, p<0.0001**** and MAHRP2/SBP1 and MAHRP2/MAHRP1 p= 0.0013**); MAHRP2/REX1 and MAHRP2/SBP1 co-location coefficient are not significantly different (unpaired t-test, p = 0.0611 ns). (F) Table showing the mean Pearson’s correlation coefficient values of REX1/SBP1/MAHRP1 versus MAHRP2-HA staining. [file Image2.tif]

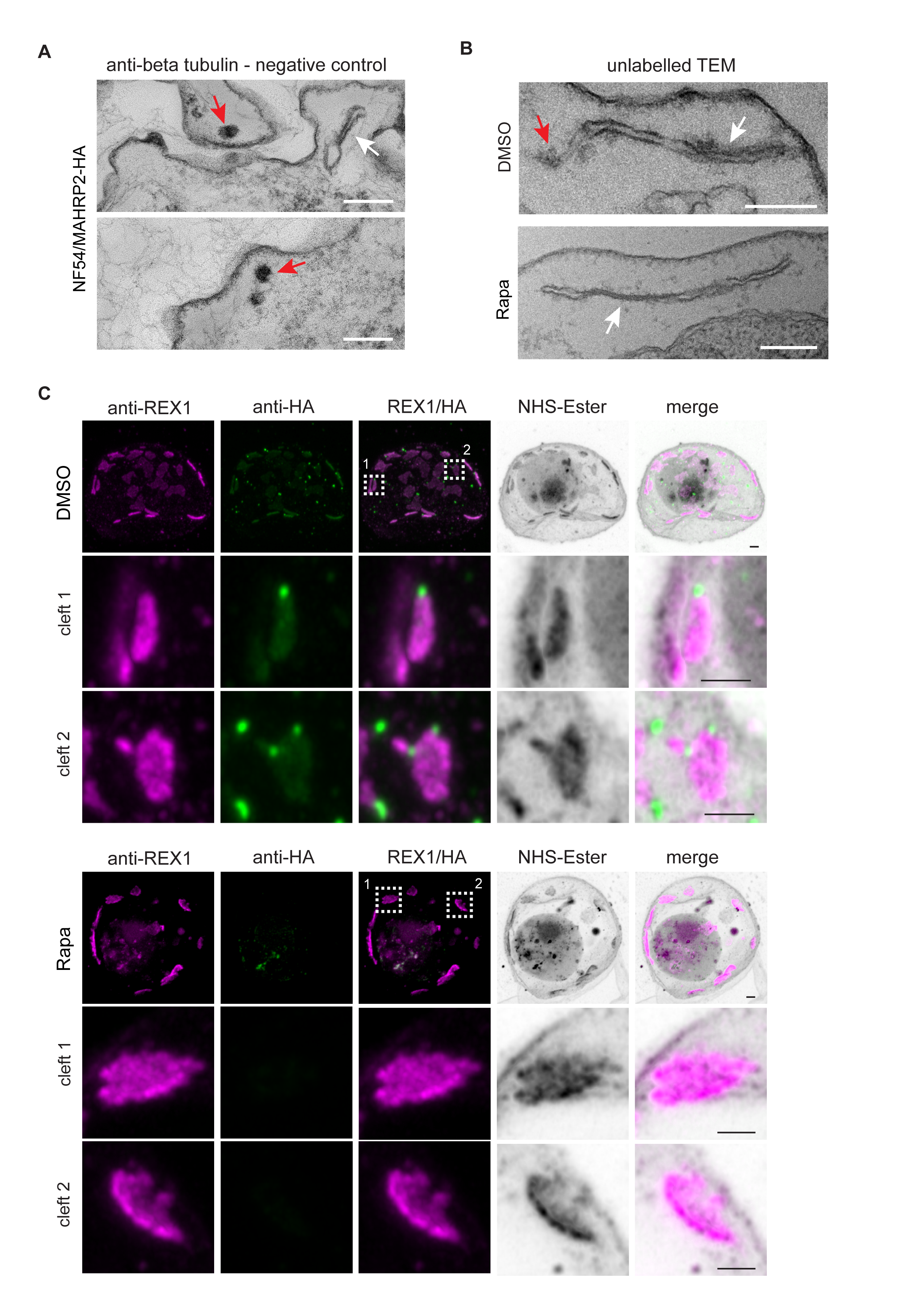

Supplement: Supplementary Figure 3 — The effect of MAHRP2 cKO on Maurer’s cleft morphology. (A) Negative control for immuno-EM. NF54-MAHRP2-HA labelled with mouse anti-beta tubulin primary raised in the same animal showing no labelling confirming absence of non-specific binding of the gold secondary antibody. Possible MAHRP2 vesicles are indicated with red arrows. Maurer’s clefts – white arrows. Scale: 200 nm. (B) Additional images for the unlabeled thin-section TEM of DMSO and Rapa treated iRBC. Maurer’s clefts are shown in both DMSO and Rapa treated iRBCs. Tethers (indicated with red arrow) were observed in DMSO treated iRBCs only. Scale: 200 nm. (C) Additional examples of expansion microscopy images of a DMSO and Rapa treated iRBC. Maurer’s clefts are labelled by anti-REX1 (magenta) and MAHRP2 labelled by anti-HA (green). NHS-Ester labels all proteins. Merged images of REX1/HA and REX1/HA/NHS-Ester are shown. Zoom images of maximum projections of z-stacks of single clefts are shown. [file Image3.tif]

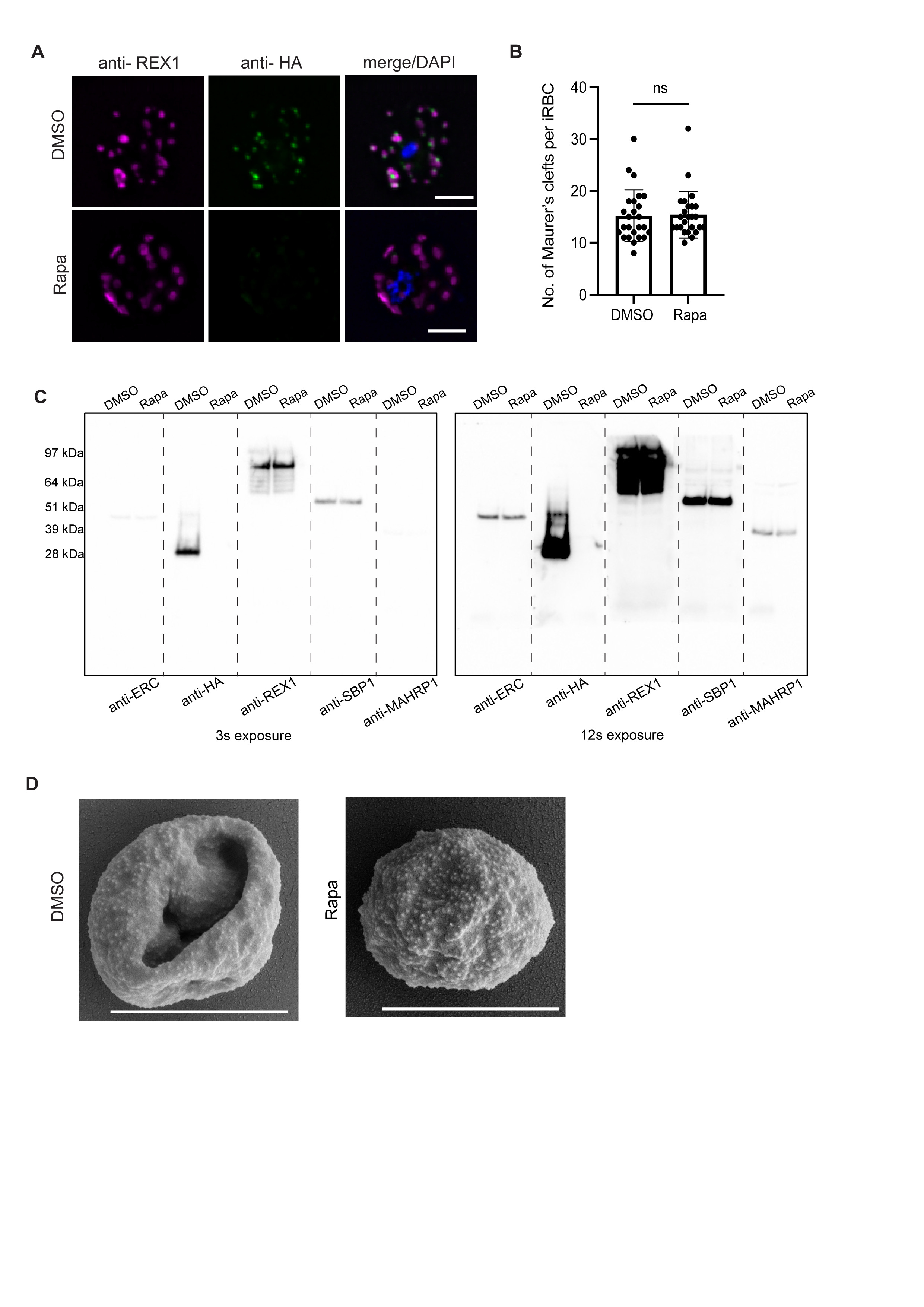

Supplement: Supplementary Figure 4 — The effect of MAHRP2 cKO on Maurer’s cleft proteins and knobs (A) Immunofluorescence imaging of DMSO and Rapa treated MAHRP2-HA cells probed with anti-SBP1 and anti-MAHRP1 to examine the Maurer’s clefts. Scale: 500 nm. (B) Bar graph showing number of Maurer’s cleft in the DMSO and Rapa treated MAHRP2-HA cells. p = 0.60 ns, n= 25 cells from 3 biological replicates. (C) Full uncut Western blots (of Figure 1D) at 3s exposure and 12s exposure showing equal loading of saponin pellets of MAHRP2-HA cells treated with DMSO or Rapa. Loss of anti-HA signal in Rapa treated sample confirms protein knockout. Anti-ERC was used as a loading control. Anti-REX1, anti-SBP1 and anti-MAHRP1 antibodies were used to examine Maurer’s cleft protein expression; no apparent difference in between the DMSO and Rapa samples. (D) Additional images of external SEM of control and cKO iRBC. Scale: 4 µm. [file Image4.tif]

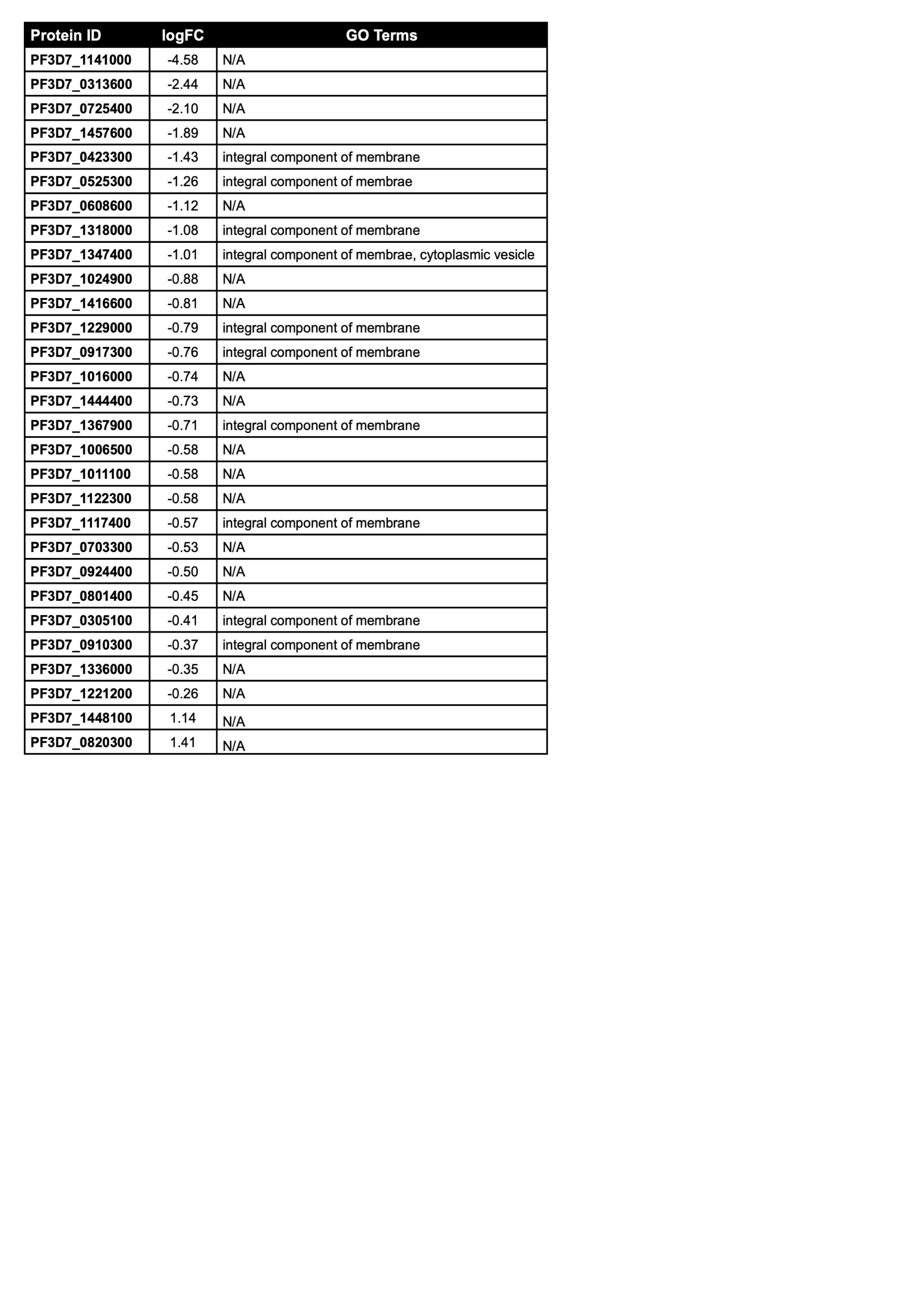

Supplement: Supplementary Figure 5 — List of uncharacterized Plasmodium falciparum proteins identified to be significantly depleted or enriched in the MAHRP2 cKO cells. log(Fold Change) and GO Terms where available are shown, N/A - not available. [file Image5.tif]

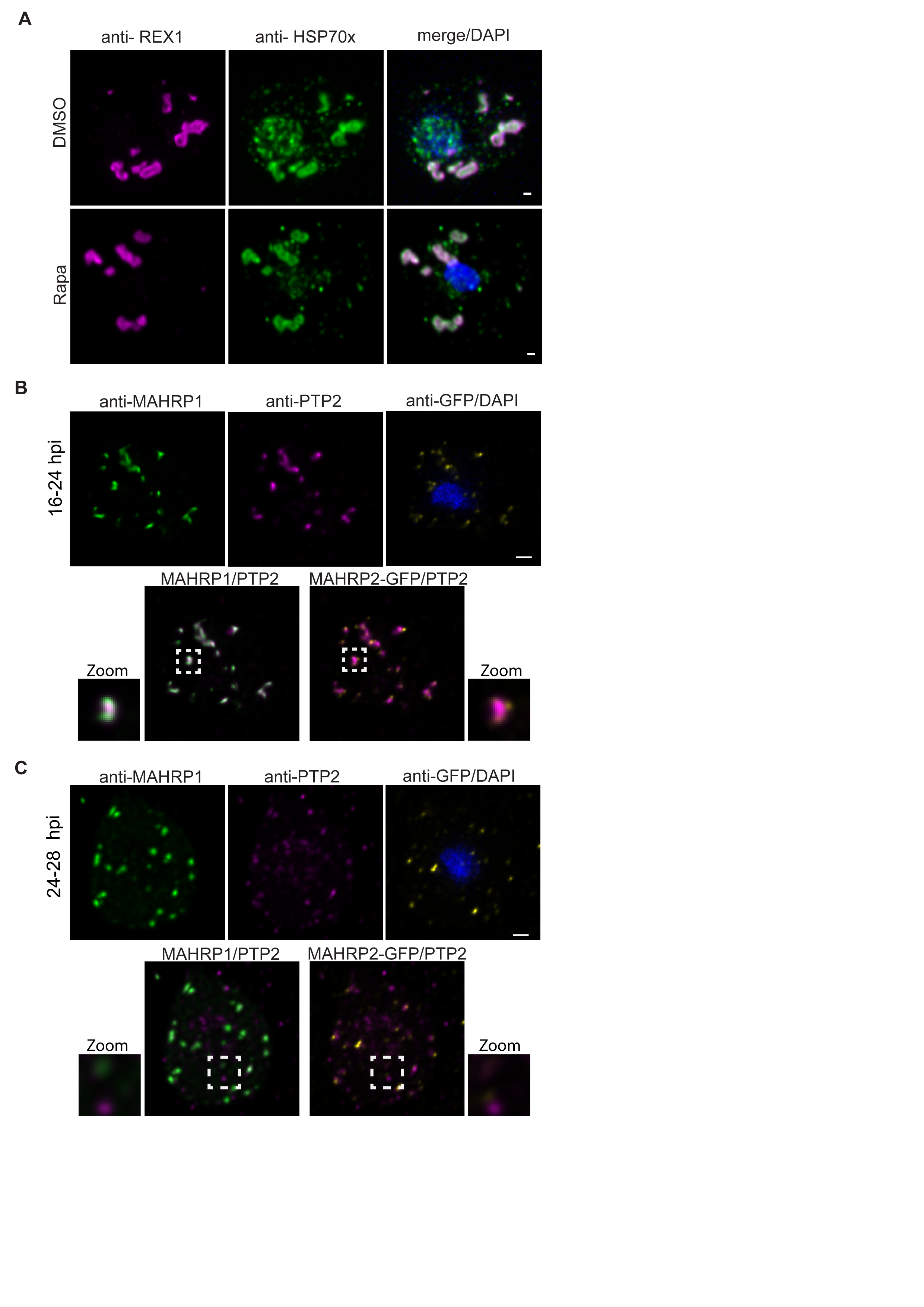

Supplement: Supplementary Figure 6 — Effect of MAHRP2 cKO on HSP70x and triple-labelling of MAHRP2-GFP (A) Super-resolution microscopy of DMSO and Rapa treated MAHRP2-HA cells (24–28 hpi) probed with anti-REX1 (magenta) and anti-HSP70x (green). A merge of the 2 channels and DAPI are shown. Scale: 500 nm. (B, C) Additional images for MAHRP2-GFP cells triple-labelled using anti-GFP (yellow), anti-PTP2 (magenta), anti-MAHRP1 (green) and the DNA label (DAPI; blue) are shown. A merge of the MAHRP1/PTP2 channels and MAHRP2-GFP/PTP2 are shown. (B) Parasites of age 16–24 hpi were imaged. Zoomed images of a single Maurer’s cleft showing that PTP2 co-locates with MAHRP1 in the inner Maurer’s cleft region and only partially co-locates with MAHRP2 in the cleft periphery. Scale bar – 500 nm. (C) Parasites of age 24–28 hpi were imaged. Zoomed images show magenta PTP2 puncta in the cytoplasm independent of Maurer’s clefts. [file Image6.tif]

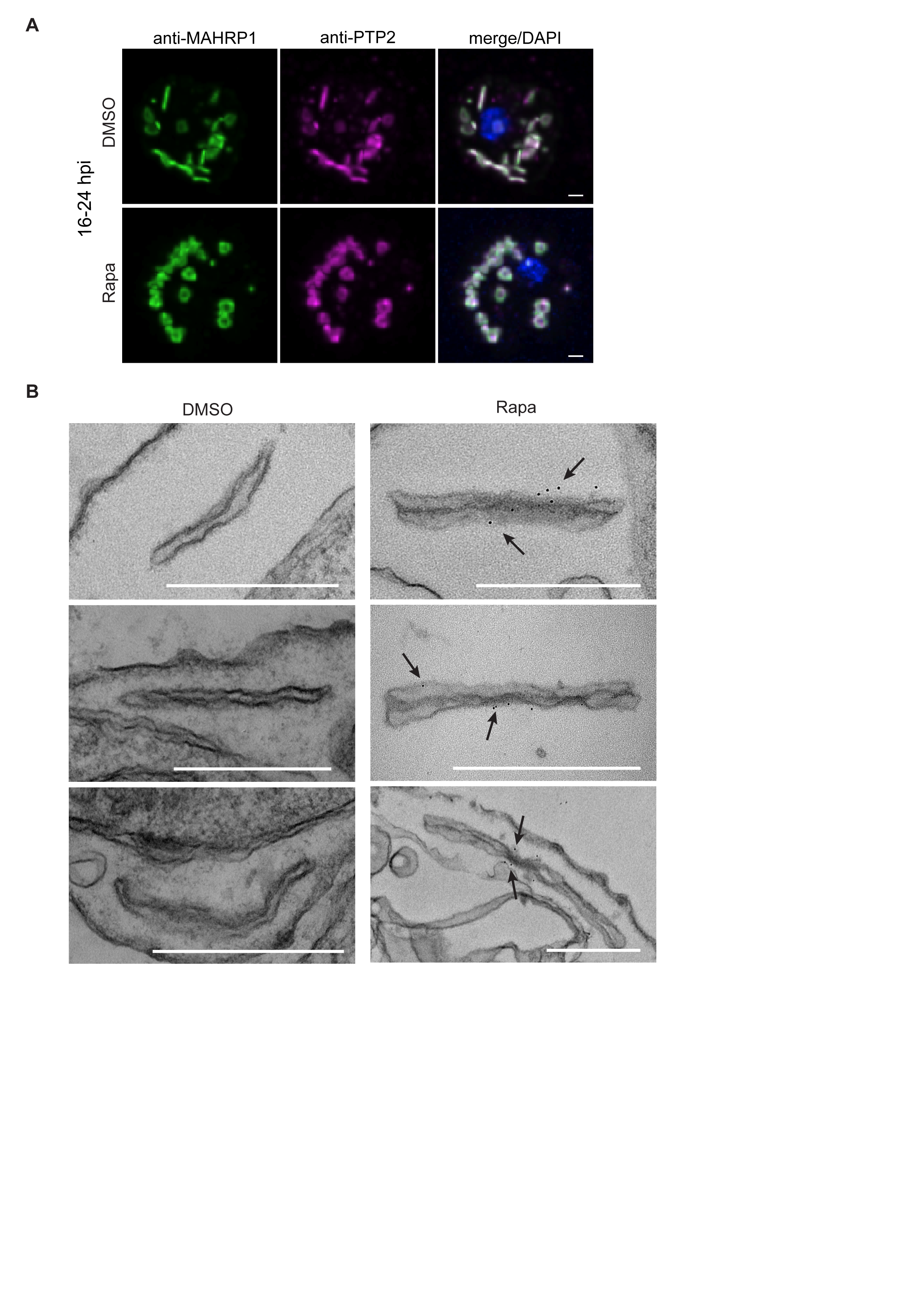

Supplement: Supplementary Figure 7 — Effect of MAHRP2 cKO on PTP2 (A) Super-resolution microscopy of 16–24 hpi DMSO and Rapa treated MAHRP2-HA cells labelled using anti-MAHRP1 (green), anti-PTP2 (magenta) and the DNA label (DAPI; blue) are shown. A merge of the 2 channels and DAPI are shown. Scale: 500 nm. PTP2 is observed to be located at the Maurer’s cleft in both DMSO and Rapa treated iRBC at this age window. (B) Additional images of DMSO and Rapa treated parasites with anti-PTP2 primary labelling and gold bead secondary labelling. MAHRP2 cKO showing abundant labelling of PTP2 (black arrows) on the periphery of Maurer’s clefts. Scale bar – 500 nm. [file Image7.tif]
