# Supplementary material for: MAHRP2 is required for tether formation and cytoadherence in Plasmodium falciparum infected red blood cells
Source: Front Cell Infect Microbiol. 2026 May 29;16:1675134. doi: 10.3389/fcimb.2026.1675134 (PMC13259744; doi:10.3389/fcimb.2026.1675134)
Supplement: Supplementary file 9 [file Table2.docx]

**Supplementary document 2_ “MAHRP2 is required for tether formation and PfEMP1 trafficking in Plasmodium falciparum infected red blood cells”**

loxP sequence – blue

3x HA sequence – green

GlmS riboswitch – grey

>MAHRP2 repair template

GATCTTcattttttttaaattatattccaaaaaaaagatatacataaattgaatgtatttttttatataaataaaaaaaaaaaaaaaaaaatattattattatttttatattatatttatttatatttttatttcacatctttatatttttgataaagaatttggaaaccaacccacaaatatagcaataaataccaaggacgccttaaagatttaatattcattaaaatattaatttttatatttatttctatttcttttttaaatataattaattgtatataattttacctaataaaaattatatacatatatatatatatatatatatatatatatatatatatatatttatatttatatatatataagtaaaattctttttctttttttttttttctttttttttttaatttttttttatttcgttttattatccttttatcaaaATGCAGCCTgtaagatataaaagataaatatatgaataaaatataagacttttgaataaatagatacatatatataATAACTTCGTATAATGTATGCTATACGAAGTTATtatatatatatatatatatatatatatatatatatatatgtatttatatatatttttttttttttagTGTCCCTACGACGTTTACAATCAAATTAACCACGTAGGAACCCATTGGGCTCAACACCTTGGTGAGCATTTGCATCACCTTGCTCACATGCACCAACATACTCCTCATGTTCATCATCATATACCTCACGTTCATACCTTAGCTCATGACAGGCCATGTATGCCAATAACCGCCTTCTTTTGTAGACACCACGAACACTGCTCATCTCACCTTATGCTTATATTCTTACTTTTGGCATTTTTCTTAGTTGTGGTATATCGTCTTTATAATGAGGTTGTGAACAGTGCAAAGACAGTTCGTATAGTTAACATAACCCCAGTTAATGAGGAACATAAAGCCGAAGCCAGTAAGGAGCAGAGTAAGTCTACATCAGATTCATCAACATCTACTCAACAGACATTAGCTGCCGCAGATTTTTCTGCAGTACCCGTACGACGTCCCGGACTACGCTGGCTATCCCTATGATGTGCCCGATTATGCGTATCCGTACGATGTTCCAGATTATGCCtaatgatagATAACTTCGTATAATGTATGCTATACGAAGTTATactagTCAGACCAGCTGTAATTATAGCGCCCGAACTAAGCGCCCGGAAAAAGGCTTAGTTGACGAGGATGGAGGTTATCGAATTTTCGGCGGATGCCTCCCGGCTGAGTGTGCAGATCACAGCCGTAAGGATTTCTTCAAACCAAGGGGGTGACTCCTTGAACAAAGAGAAATCACATGATCTCTCGAGGGATATGGCAGCTTAATGTTCGTTTTTCTTATTTATATATTTATACCAATTGATTGTATTTATAACTGTAAAAATGTGTATGTTGTGTGCATATTTTTTTTTGTGCATGCACATGCATGTAAATAGCTAAAATTATGAACATTTTATTTTTTGTTCAGAAAAAAAAAACTTTACACACATAAAATGGCTAGTATGAATAGCCATATTTTATATAAATTAAATCCTATGAATTTATGACCATATTAAAAATTTAGATATTTATGGAACATAATATGTTTGAAACAATAAGACAAAATTATTATTATTATTATTATTTTTACTGTTATAATTATGTGTCTCCTTCAATGATTCATAAATAGTTGGACTTGATTTTTAAAATGTTTATAATATGATTAGCATAGTTAAATAAAAAAAGTTGAAAAATTAAAAAAAAACATATAAACACAAATGATGGTTTTTCCTTCAATTTCgatatcATATAGCAATCGTATTAGCAGGTAAATGAGCAAGGTTAATAAAAAAATGGTAAAAAAAAAAAATTGGGGAAAAATAAACAAGGTGTCTTTATCTACACTTTGGCTTTACAATGTATAATTTATATGCATACAAAAAAATTTTGATAAAATTGTAAAAAGGTGAATAAAATAACATTGTAACAGTAGTAGATAGTAAAGGGAAGGTGTTGCTCAAATAGTGTCGAAACAAAACTGGCATAAAGAAAAATTGATATTGAGCAGAGGATATGCGCATAATGGTATTTTGTTTGTTTGTTTGTTTTCTCATTTTTTGAGACAGCTCAATTCTTTATGTCCACAACATCATCGGACTTTTCTTCTTCAGGGTAGGCGGCCGCAGGTCCAATTTATAGAAACAAAATATATACTTGTATAATTTTATTTTTTTATATAAATCATTACATATATAATTATACAATATTTTTTCTAAGAGATAATTATATATTAATATATATAAAAAAAGGTGTTTTTTTTTTTTTTTTTTATTTTTATTTTTATTTTATGGTAATATTTTATTTTCCTTATTTTATAAATTATATTAGTTTATATGTGATTAATTTTATATATTATCAATTTATATATTTTTAAATGCTTACTTAATTATCTTTTTTTTTTTTTTTTTTTTTTTTTCCCCTCTTTTTATATTAATTTATTTTTGAAAAAATTGATATATATATATATATATAATATATATATATACATGTAGTAGTATTAAACAATGTATAATATATATAAATAATATATTTATATATTTCATTTCAATTTTAATTTTTTTTGGTTTTTTTTTTTTTTCTTTTTGTCATATTTAAAAAAAATTATATTCATATAAGTTATGCATTTTTTATAAACATTATTCAATATATGTATAATATAATATATATATATATATTAATGTATTATTCCAATGTGCATGATAAAAGAAAAAAATAATATTTATAAAAAAAAAGAAAAATAAAACAAAAAAAGAAAAAAAAAAAAAAAAAAAAAAAAATACAAAAATAAATAATATAATTTATAATTATATATTCTTGTCACAATAAAAATATATATATATATATATATATTTATAATATGTATATTTTAAACTAGAAAAGGAATAACTAATATTTTATTTATTATCATTCAAGATTTATATTTTATAATAATAAATACCTAATAGAAATATATCAGGATCCAAAAAATGCATGGTTCGCTAAACTGCATCGTCGCTGTGTCCCAGAACATGGGCATCGGCAAGAACGGGGACTACCCCTGGCCACCGCTCAGGAACGAATTTAGATATTTCCAGAGAATGACCACAACCTCTTCAGTAGAAGGTAAACAGAATCTGGTGATTATGGGTAAGAAGACCTGGTTCTCCATTCCTGAGAAGAATCGACCTTTAAAGGGTAGAATTAATTTAGTTCTCAGCAGAGAACTCAAGGAACCTCCACAAGGAGCTCATTTTCTTTCCAGAAGTCTAGATGATGCCTTAAAACTTACTGAACAACCAGAATTAGCAAATAAAGTAGACATGGTCTGGATAGTTGGTGGCAGTTCTGTTTATAAGGAAGCCATGAATCACCCAGGCCATCTTAAACTATTTGTGACAAGGATCATGCAAGACTTTGAAAGTGACACGTTTTTTCCAGAAATTGATTTGGAGAAATATAAACTTCTGCCAGAATACCCAGGTGTTCTCTCTGATGTCCAGGAGGAGAAAGGCATTAAGTACAAATTTGAAGTATATGAGAAGAATGATTAAaagcttATTTAATAATAGATTAAAAATATTATAAAAATAAAAACATAAACACAGAAATTACAAAAAAAATACATATGAATTTTTTTTTTGTAATCTTCCTTATAAATATAGAATAATGAATCATATAAAACATATCATTATTCATTTATTTACATTTAAAATTATTGTTTCAGTATCTTTAATTTATTATGTATATATAAAAATAACTTACAATTTTATTAATAAACAATATATGTTTATTAATTCATGTTTTGTAATTTATGGGATAGCGATTTTTTTTACTGTCTGTATTTTTCTTTTTTAATTATGTTTTAATTGTATTTTATTTTTATTATTGTTCTTTTTATAGTATTATTTTAAAACAAAATGTATTTTCTAAGAACTTATAATAATAATAAATATAAATTTTAATAAAAATTATATTTATCTTTTACAATATGAACATAAAGTACAACATTAATATATAGCTTTTAATATTTTTATTCCTAATCATGTAAATCTTAAATTTTTCTTTTTAAACATATGTTAAATATTTATTTCTCATTATATATAAGAACATATTTATTAAATCTAGAATTCCAAATAAACCATGTAGGAACTCATTGGGCTCAACATTTAGGAGAACACTTACATCATTTAGCACATATGCATCAACATACTCCACATGTACATCATCACATTCCACATGTGCATACGCTAGCTCATGATAGGCCTTGTATGCCAATAACTGCTTTCTTTTGTCGACATCATGAACATTGTAGTTCCCATTTAATGTTAATCTTTTTATTATTGGCTTTCTTCCTAGTAGTAGTTTATAGATTATATAACGAGgtaagttattattaacatatataagttttttattcaatacatattatatatatatatatattagctgattttattgcattatatattatatatgtacctttattccttttttatttttcagGTTGTTAATTCAGCTAAAACTGTACGTATTGTAAATATAACACCAGTAAATGAGGAACATAAGGCTGAAGCTAGTAAGGAACAATCAAAATCAACAAGTGATTCTTCTACTAGTACACAACAAACATTAG
